# Supplementary material for: Inferring language dispersal patterns with velocity field estimation
Source: Nat Commun. 2024 Jan 2;15:190. doi: 10.1038/s41467-023-44430-5 (PMC10761963; doi:10.1038/s41467-023-44430-5)
Supplement: Supplementary file 1 — Supplementary Information [file 41467_2023_44430_MOESM1_ESM.pdf]

Supplementary Information

Inferring language dispersal patterns with velocity field estimation

Authors and Affiliations

Sizhe Yang[1], Xiaoru Sun[2, 3], Li Jin[1, 2 *], Menghan Zhang[4,5 *]

[1] State Key Laboratory of Genetic Engineering, Center for Evolutionary Biology, and Collaborative Innovation Center for Genetics and Development, School of Life Sciences, Fudan University, Shanghai, 200438, China

[2] Human Phenome Institute, Fudan University, Shanghai, 200438, China

[3] Ministry of Education Key Laboratory of Contemporary Anthropology, Department of Anthropology and Human Genetics, School of Life Sciences, Fudan University, Shanghai, 200438, China

[4] Institute of Modern Languages and Linguistics, Fudan University, Shanghai, 200433, China

[5] Research Institute of Intelligent Complex Systems, Fudan University, Shanghai, 200433 China

* Menghan Zhang, Li Jin

Email: mhzhang@fudan.edu.cn, lijin@fudan.edu.cn

Contents

SUPPLEMENTARY NOTES **4**

SUPPLEMENTARY SECTION 1: THE RATIONALE OF THE LVF 4

1.1 The consistency and inconsistency between LVF and phylogeographic approach *4*

1.1.1 The consistency between LVF and phylogeographic approach 4

1.1.2 The inconsistency between LVF and phylogeographic approach 4

1.2 Parametric definition and estimation of prestige *6*

1.2.1 Definition of the prestige 6

1.2.2 Estimation of the prestige 7

1.3 Language dispersal centre inference *8*

1.3.1 Strategies for dispersal centre inference 8

1.3.2 Assessing the influence of heterogeneity of language spatial distribution 8

SUPPLEMENTARY SECTION 2: SIMULATED VALIDATIONS FOR LVF 9

2.1 Simulation design *9*

2.2 Simulation results *10*

2.2.1 The evaluation of effectiveness 10

2.2.2 The evaluation of robustness 10

2.2.3 The ranges of parametric settings 11

SUPPLEMENTARY DISCUSSION **12**

SUPPLEMENTARY SECTION 1: INTRODUCTION OF THE FOUR LANGUAGE FAMILIES AND GROUPS WORLDWIDE 12

1.1 The reasons for selecting the four language families and groups *12*

1.2 Indo-European languages *13*

1.3 Sino-Tibetan languages *13*

1.4 Bantu languages *14*

1.5 Arawak languages *15*

SUPPLEMENTARY SECTION 2: THE AGE-AREA HYPOTHESIS FOR DIVERSITY APPROACH 15

SUPPLEMENTARY SECTION 3: THE MOTIVATIONS AND DISCUSSIONS FOR PROPOSING THE LVF 16

3.1 The limitations of the phylogeography *16*

3.2 The motivations for proposing the LVF *16*

3.3 The advantages of the LVF *17*

3.4 The prospects of the LVF *17*

SUPPLEMENTARY METHODS **19**

SUPPLEMENTARY SECTION 1: MATHEMATICAL FORMULAS AND DERIVATIONS FOR THE LVF 19

1.1 The calculation of the velocity field *19*

1.1.1 Principle for the parametric estimation of prestige in the dynamic model 19

1.1.2 The reconstruction of the past states for linguistic traits using a dynamic model 21

1.1.3 Velocity field establishment 23

1.2 The projection of the velocity field *24*
1.2.1 PCA projection of the velocity field........ 24
1.2.2 Kernel projection of the velocity field 24
1.2.3 Spatial smoothing for the velocity field 27
1.2.4 Grid smoothing for the velocity field........ 28
1.3 Language dispersal centre inference *28*
1.3.1 Inferring the language dispersal centre 28
1.3.2 Inferring the Standard Deviation of the language dispersal centre........ 29
1.3.3 Comparison with the diversity approach........ 30
1.4 The validation for the robustness of the LVF *31*
1.4.1 Validating the efficiency of the mode-value imputation........ 31
1.4.2 Validating the robustness of the LVF against the setting of parameter k 32
1.4.3 Validating the robustness of the LVF against the setting of parameter λ 33
1.4.4 Validating the robustness of the LVF against the setting of parameter m 33
SUPPLEMENTARY REFERENCES **35**
SUPPLEMENTARY TABLES **42**
SUPPLEMENTARY FIGURES **45**

Supplementary Notes

Supplementary Section 1: The rationale of the LVF

1.1 The consistency and inconsistency between LVF and phylogeographic approach

1.1.1 The consistency between LVF and phylogeographic approach

LVF shares the same theoretical foundation as the phylogeographic approach. They both infer language dispersal patterns by investigating the diachronic evolution of linguistic traits that shape the observed linguistic relatedness (Supplementary Fig. 1). The feasibility of these two approaches is guaranteed by the correlation between linguistic relatedness and language geography [1-3]. To be specific, languages sharing closer geographic locations usually exhibit greater linguistic relatedness. It can be attributed to either vertical divergence or horizontal contact. From the divergence perspective, the closer geographic locations among languages indicate that after diverging from their common ancestor, their geographic dispersal may have only lasted for a shorter period [1]. During such a shorter period, the linguistic traits among these languages would have only accumulated fewer variations that cause their higher linguistic relatedness. From the contact perspective, the languages situated within the closer geographic range would exhibit a higher likelihood of contacting each other. This intensive contact may facilitate the languages to borrow linguistic traits from other neighbouring languages, which enhances their linguistic relatedness. Accordingly, the differentiations among the geographic locations of languages consistently align with the variations in their linguistic traits that can be attributed to either divergence or contact. If we can manage to illustrate the diachronic evolution of linguistic traits in languages, we can correspondingly deduce their geographic dispersal history.

1.1.2 The inconsistency between LVF and phylogeographic approach

Both LVF and phylogeographic approaches entail two identical major steps to infer the language dispersal pattern. The first is to establish the diachronic evolutionary trajectories of linguistic traits that can explain the formation of the observed linguistic relatedness. The second is to transform such diachronic evolutionary trajectories of linguistic traits into language dispersal trajectories, according to the correlation between linguistic relatedness and language geography. However, LVF and the phylogeographic approach implement different strategies to carry out these two major steps.

The depiction of diachronic evolutionary trajectories of linguistic traits that shape observed linguistic relatedness. The phylogeographic approach leverages the phylogenetic tree to depict the diachronic evolutionary trajectories of linguistic traits that shape observed linguistic relatedness [2-5]. These evolutionary trajectories are mirrored by the branching patterns within the phylogenetic tree (Supplementary Fig. 1). Such branching patterns outline the evolutionary directions of linguistic traits in languages after they diverged from

their most recent common ancestor (MRCA). To be specific, the branching patterns render how the linguistic traits in each language evolve from their ancestor states to their current states. The shorter branch linking two languages embodies fewer trait variations between them, resulting in their higher linguistic relatedness [6,7]. However, the phylogenetic tree can only explain the partial linguistic relatedness attributed to vertical divergence. Accordingly, the diachronic evolutionary trajectories of linguistic traits depicted by the phylogenetic tree may not be able to adequately interpret the formation of observed linguistic relatedness.

In contrast to the phylogenetic tree, LVF utilizes the velocity field to outline the diachronic evolutionary trajectories of linguistic traits that shape observed linguistic relatedness (Supplementary Fig. 1). This velocity field can capture the attributions of both vertical divergence and horizontal contact in shaping observed linguistic relatedness. To be specific, this velocity field is established in a two-dimensional PC space in which each velocity vector is attached to a language within that PC space. The shorter Euclidean distances among languages imply their higher linguistic relatedness due to either divergence or contact. The velocity vector of a language visualized as an arrow roughly reflects the evolutionary directions of its linguistic traits, which functions similarly to the branch within the phylogenetic tree. To be specific, the vector direction of language can render how the linguistic traits in this language evolved from their past states to their current states within the PC space. With these vector directions, a collection of trajectories can thus be visualized to outline the diachronic evolutionary trajectories of linguistic traits that shape the observed linguistic relatedness. These trajectories function similarly to the trajectories reflected by the branching patterns within the phylogenetic tree but additionally capture the attribution of horizontal contact.

The geographic projection of evolutionary trajectories of linguistic traits that shape observed linguistic relatedness. To transform the evolutionary trajectories of linguistic traits into language dispersal trajectories, the phylogeographic approach projects the phylogenetic tree into geographic space based on the correlation between linguistic relatedness and language geography (Supplementary Fig. 1) [1-3,8]. After the geographic projection, the branching patterns within the phylogenetic tree are considered as the dispersal trajectories. To be specific, each branch projected into the geographic space is regarded as a segment of the entire dispersal trajectories. This projection is achieved by applying the random walk process to the phylogenetic tree. With the random walk process, the shorter branch between two languages, indicating their higher linguistic relatedness, would be transformed into a shorter geographic trajectory between them. Consequently, the linguistic relatedness between two languages is reframed in terms of the length of their dispersal trajectory within the geographic space. Under this circumstance, we can regard the geographic projection of the phylogenetic tree as the spatial adjustment of its branching patterns within the geographic space. It ensures that the formations of linguistic relatedness and geography of languages can be both explained by the branching patterns within the phylogenetic tree.

In contrast to the random walk process, LVF utilizes the kernel projection proposed by La Manno et al. [9] to project the velocity field from PC space into geographic space (Supplementary Fig. 1). Based on the

correlation between linguistic relatedness and language geography, the kernel projection seeks the velocity vector in the geographic space, ensuring its correlation with language geography matches closely to its correlation with linguistic relatedness. After the kernel projection, the directions of velocity vectors delineate from where these languages diffused into their current locations. Consequently, the velocity field composed of velocity vectors outlines a set of dispersal trajectories. Under this circumstance, we can regard the geographic projection of the velocity field as the spatial adjustment of its velocity vectors within the geographic space. It ensures that the formations of linguistic relatedness and geography of languages can be both explained by the velocity vectors.

1.2 Parametric definition and estimation of prestige

The estimation of the prestige parameter stands as a pivotal stride in implementing our dynamic model. A reasonably defined prestige parameter can improve the explanatory power of the dynamic model [10]. Typically, the parametric estimation of a dynamic model necessitates a sequential array of data points collected at various temporal junctures for robust model fitting. However, in most linguistic studies, it is common that the available data points are collected at the same current time [11,12]. Accordingly, the lack of time-series data points hampers the parametric estimation of our dynamic model and further impedes the modeling of the language dynamics. Noting these, we proposed a principle for the parametric estimation of the prestige in our dynamic model, which can only rely solely on the data points collected at the same current time (See detailed mathematical formulas in Supplementary Methods section 1.1.1).

1.2.1 Definition of the prestige

Definition of the prestige. Prior to the parametric estimation, we embarked on the definition of the prestige of each state in a linguistic trait. In the original AS model, prestige is an abstract parameter that reflects the social or economic opportunities afforded to its speakers. This abstract parameter has been redefined as the inheritance rate of a language in its subsequent research [10]. To be specific, the offspring of the language speakers wielding higher prestige exhibited a heightened probability of retaining their parental tongue, thereby bolstering the prevalence of this language among the speakers. In contrast, the offspring of speakers endowed with lower prestige may inherit their parental language with a lower likelihood, potentially culminating in the gradual decline of that language over successive generations. In analog to this redefinition of prestige, the prestige of a specific state in a linguistic trait is here defined as the probability of this linguistic trait maintaining in this state after a unit of time.

Definition of a unit of time. Given that we often have limited knowledge regarding the precise origin time and trait states of past languages, we thus define a unit of time as one generation, which serves as a dimensionless time indicator representing the period during which the linguistic traits in language accumulate one mutation. This definition of the unit of time in our study is identical to the definition in the phylogenetic

tree where no exact time calibrations have been made (hereafter non-time-calibrated phylogenetic tree). To be specific, in a non-time-calibrated phylogenetic tree, the branch length between a parent node and a child node (we refer to a node as a language for convenience hereafter) represents the time during which the child language has evolved from its parent language. This branch length is typically represented by the number of mutations that occurred in linguistic traits during the evolution of the child language from its parent language. This is due to that the longer evolutionary time of a language results in more mutations being accumulated in linguistic traits [13,14]. Under this circumstance, a unit of time is defined as the period during which the linguistic traits of language undergo one mutation.

1.2.2 Estimation of the prestige

To estimate the value of prestige, we introduced a principle of parametric estimation derived from the DNA substitution model for genetic evolution proposed by Felsenstein [15,16]. This substitution model rests upon the Poisson process, allowing each base of the genetic site within the DNA sequence to undergo transitions to other bases at arbitrary times with a heterogeneous rate during the genetic evolution. The nature of the Poisson process harmonizes with our model's assumptions that each linguistic trait can experience multiple transitions between gain and loss, exhibiting a heterogeneous rate throughout linguistic trait evolution. It also agrees with the linguists' institutions that each linguistic trait has its distinct evolutionary process. Therefore, we harnessed the Poisson process to simulate the gain and loss of the linguistic trait and calculate the prestige parameters of its different states.

The DNA substitution model introduced by Felsenstein is widely employed in the modeling studies of the DNA evolutionary process based on a given phylogenetic tree. This model assumes that the parameter of the Poisson process referred to as the transition probability corresponds to the empirical frequency of each base of a given genetic site within the population (See the mathematical definition of this parameter in Supplementary Methods section 1.1.1) [16]. This parametric setting of the transition probability can better interpret the formation of the current distribution of the base in each genetic site. With this parameterization, the Poisson process serves as a useful tool for depicting transitions between various genetic bases and subsequently reconstructing genetic evolution. In this study, following the DNA substitution model, we set the frequency of each state for each linguistic trait in the language samples as the transition probability. Through the application of the Poisson process, we calculated the prestige values of different states using the Poisson process (See detailed mathematical formulas in Supplementary Methods section 1.1.1). Although our parametric estimation principle does not necessitate phylogenetic tree reconstruction of the languages, its conceptual and methodological bases are still derived from the phylogenetic methods.

1.3 Language dispersal centre inference

1.3.1 Strategies for dispersal centre inference

To infer the language dispersal centre, we designed a strategy (hereafter radiative strategy) that is founded upon the grid-smoothed velocity field in the geographic space. We postulated that the languages around the dispersal centre should spread outwards in all directions. Given that the velocity vectors reflect the language dispersal directions, the velocity vectors surrounding the dispersal centre should emanate radially from this centre. Therefore, the language dispersal centre should correspond to the grid point encircled by the grid-smoothed velocity vectors which exhibit the strongest outwards radiative pattern. The intensity of this pattern is assessed through the measurement of the variance of the directions of the grid-smoothed velocity vectors surrounding each grid point. The grid point displaying the highest variance of directions of neighbouring velocity vectors is considered a potential dispersal centre.

However, in practice, this strategy may not always function well. The reason is that the language dispersal pattern could also appear as a chained topology whose dispersal trend generally points in the same direction [17]. Such a trend could be attributed to geographic or ecological constraints such as oceans, mountains, and islands [18]. Noting these, we also proposed a simple strategy (hereafter chained strategy) to infer the language dispersal centre of the chained dispersal pattern. Firstly, we calculated the convex hull of the language samples based on their geographic coordinates. Secondly, we calculated the average of the grid-smoothed velocity vectors within the geographic space. The direction of this average grid-smoothed velocity vector signifies the predominant dispersal direction of the language samples. Within the chained topology, we posit that the dispersal centre is the geographic location of the language sample situated at the border of the convex hull, whose velocity vector should exhibit the highest correlation with the average grid-smoothed velocity vector.

It is noted that the chained strategy is just a simple criterion that may not align perfectly with the linguistic reality. The dispersal centre identification of chained dispersal topology necessitates further detailed investigation. However, this chained strategy is worth trying when the velocity vectors of one language family or group manifest a significant chained dispersal pattern. It is noted that employing and improving the chained strategy is beyond the scope of our current work since the radiative strategy is observed to function effectively in both simulated and empirical applications. Nevertheless, we still look forward to addressing the challenge of effectively identifying the dispersal centre of chained dispersal topology in our future work.

1.3.2 Assessing the influence of heterogeneity of language spatial distribution

It is noted that any spatial analysis methodology is inevitably affected by the spatial distribution of samples. Accordingly, we next assessed whether the dispersion of the language spatial distribution could influence the estimation of the LVF. Firstly, we calculated the Standard Deviation (SD) values of the

coordinate in terms of longitude and latitude of the inferred dispersal centre in each language family or group, using the traditional Jackknife resampling approach [19] (See detailed mathematical formulas in Supplementary Methods section 1.3.2). We found that the difference among the SD values could be attributed to the heterogeneity of the geographic distributions of language samples in different language families and groups (Supplementary Table 2). More precisely, greater geographic dispersion of language samples introduced increased uncertainties in the estimation of dispersal centres. Subsequently, we carried out the linear regression analysis and the result renders the high association between the SD value and the geographic dispersion of language samples (Supplementary Fig. 11). This outcome signifies that the dispersal centre estimated by LVF indeed can be affected by the spatial heterogeneity of language samples distributed across the geographic space.

Supplementary Section 2: Simulated validations for LVF

2.1 Simulation design

Using four realistic cases of agricultural languages around the world, we have demonstrated that the language dispersal patterns yielded by our LVF can be favored by the known genetic and archaeological evidence. However, the effectiveness and robustness of LVF still warrant detailed validations. Given the scarcity of knowledge about the true dispersal patterns, it is hard to evaluate the performance of LVF through empirical datasets. Noting these, we undertook the rigorous simulated validations for the LVF, focusing specifically on dispersal centre inference. These simulated validations leveraged 1,000 simulated linguistic datasets provided by Wichmann and Rama (2021) [20]. Furthermore, we also conducted comprehensive comparisons between the performance of LVF and the prevailing phylogeographic approach based on these 1,000 simulated datasets.

Each simulated dataset encompasses 20 language samples characterized by identical 306 binary-coded traits which are generated by a specific phylogenetic tree. The coordinates of the simulated language samples in each simulated dataset are generated by the random walk model. To be specific, these coordinates are simulated by applying the random walk model to this language phylogenetic tree which has been assigned a randomly selected dispersal centre. It is noteworthy that the 1,000 simulated datasets share consistent linguistic trait values while exhibiting variations in the coordinates of the language samples. Remarkably, the true coordinate of the dispersal centre is already known for each simulated dataset. Accordingly, we validated the effectiveness of the velocity field estimation approach by examining the difference between the true and inferred coordinates of the dispersal centre using the Wilcoxon rank-sum test.

To infer the language dispersal centre, a pivotal step is to place the grid points at a certain interval on the geographic area covered by the language samples. It is accomplished by the grid smoothing approach proposed by La Manno et. al [9]. They suggested that the number of grid points should be chosen depending on the

geographic range of the language distribution [9]. However, the geographic ranges of the language distributions in 1,000 simulated linguistic datasets manifest considerable differences. Notably, certain datasets exhibit language distributions spanning extensive longitudinal and latitudinal degrees, whereas others are confined to more limited spans. Therefore, the number of grid points can be quite different among simulated datasets if the interval between grid points is fixed. It thus would lead to incomparability among the dispersal centres inferred from different simulated linguistic datasets.

Noting these, we set the number of the grid points as a constant when applying the LVF to 1,000 simulated datasets. Next, we examined the effectiveness of the LVF under different settings of the number of grid points. Furthermore, we validated the effectiveness of LVF against different settings of other parameters. To be specific, they are the k-nearest neighbours for data conversion, the mutation rate of the Poisson process for parametric estimation of the dynamic model, and the reconstruction time for calculating the velocity field. Additionally, to examine the robustness of the LVF against different settings of those parameters, we conducted the cosine similarity to examine the similarity between the velocity fields calculated from different parametric settings.

2.2 Simulation results

2.2.1 The evaluation of effectiveness

For each simulated dataset, we varied across different values of the number of the nearest neighbours k (k = 2, 4, 6, …, and 18), the mutation rate of Poisson process λ (λ = 0.1, 0.5, 1, 5, and 10), and reconstruction time m (m = 1, 3, 5, 7, and 9) when applying the LVF. Subsequently, we deduced the coordinate of the language dispersal centre using the LVF and then calculated the differences in terms of both longitude and latitude from the true coordinate of the dispersal centre respectively. These differences denote the estimated errors of the LVF in inferring the language dispersal centre in terms of the longitude and latitude. Based on 1,000 simulated datasets, we obtained the distributions of the estimated errors under different parametric settings (Supplementary Fig. 2). The results of the Wilcoxon rank-sum test revealed that the inferred coordinates of the language dispersal centres under different parametric settings were not significantly different from the true one in terms of the longitude and latitude respectively (Supplementary Fig. 2). It confirms the effectiveness of the LVF in inferring the language dispersal pattern.

2.2.2 The evaluation of robustness

For each simulated dataset, we varied across the different values of the number of the nearest neighbours k (k = 2, 4, 6, …, and 18), the mutation rate of Poisson process λ (λ = 0.1, 0.5, 1, 5, and 10), and reconstruction time m (m = 1, 3, 5, 7, and 9) when applying the LVF. Subsequently, we calculated the average cosine similarity among the velocity vectors within different velocity fields under different parametric settings. This average cosine similarity serves as a comprehensive metric to quantify the overall similarity among different velocity

fields. The calculation of the average cosine similarity follows the procedure in Supplementary Methods section 1.4. Based on 1,000 simulated datasets, we obtained the distributions of the average cosine similarities between velocity fields in both high-dimensional and two-dimensional space estimated from different parametric settings (Supplementary Fig. 3). The results of the Wilcoxon rank-sum test showed that there was no significant difference among the velocity fields in either high-dimensional or two-dimensional space estimated from different parametric settings (Supplementary Fig. 3). It indicates the robustness of the LVF in inferring the language dispersal pattern.

2.2.3 The ranges of parametric settings

Up to now, we have validated the effectiveness and robustness of the LVF under different parametric settings. To facilitate the empirical application of the LVF, we next devoted to providing rational ranges of the parametric settings for the application of the LVF. Here, we adopted the great-circle distance [21] (the shortest distance between two points on the surface of a sphere) as a comprehensive index of the estimated error to measure the difference between the true and inferred coordinates of the language dispersal centre under each specific parametric setting. To gain a comprehensive understanding of the overall estimated error across 1,000 simulated datasets, we computed both the mean and median of these estimated errors under different parametric settings (Supplementary Fig. 12).

Our analysis revealed that the mean and median of 1,000 estimated errors were robust under different settings of mutation rate and reconstruction time. In contrast, both the mean and median of 1,000 estimated errors demonstrated a decreasing trend with an increase in the number of the grid points, while an increasing trend was observed as the number of the nearest neighbours increased. To be specific, they exhibited a trend of rapid increase when the number of the nearest neighbours was larger than 8 (accounts for 40% of the sample size of each simulated dataset). Additionally, they exhibited a trend of rapid decrease when the number of grid points was larger than 100.

According to these observations, we suggest that the number of grid points for the grid-smoothing approach should be at least 100; the number of the nearest neighbours for data conversion should be no more than 40% of the sample size of the language dataset; the mutation rate in the Poisson process can be set arbitrarily from 0.1 to 10, and the reconstruction time for calculating velocity field can also be set arbitrarily from 1 to 10.

Supplementary Discussion

Supplementary Section 1: Introduction of the four language families and groups worldwide

1.1 The reasons for selecting the four language families and groups

Rising about 10,000 years ago, the Neolithic Revolution (also known as the Agriculture Revolution) brought huge cultural transformation (e.g. transition from the hunter-gatherer society to agricultural society) and tremendous technological progress (e.g. animal and plant domestication) into the human populations [22]. It made an increasingly large population possible which drove the substantial population movements in human prehistory [23]. The massive population movements also could motivate the expansion of worldwide languages. In this study, we aimed to investigate the alignment of the demic diffusions, Neolithic and agricultural culture spreads, and language dispersals in human prehistory.

Accordingly, the primary objective of our study is to examine the spatial alignment among language dispersal, demic diffusion, and Neolithic/Agricultural culture spread in human prehistory. Therefore, the language cases utilized in our study are expected to fulfil the following criteria. Firstly, the language case should have a possible association with the origin and development of ancient agriculture. Secondly, the demic or cultural diffusions in the specific geographic areas where these languages are spoken should be supported by corresponding genetic or archaeological evidence. Thirdly, the language cases are preferably renowned cases with sufficient language samples that have been rigorously investigated in previous phylogenetic research. More importantly, the lexical items in these language cases should have been carefully collated and well coded into cognate sets that meet the standard of computational linguistics. With these criteria, we hope that the empirical cases can better serve our paper's primary objective and make our estimated results more acceptable to the broad range of audiences.

Noting these, we selected the four language families and groups that are closely associated with the developments of ancient agriculture or Neolithic cultures around the world. These four language families and groups are the hot research objects in linguistics, which have almost covered all the inhabited continents around the world. In short, the spread of the Indo-European languages is regarded as driven by the expansion of Anatolia farmers from the Fertile Crescent [2,24]. For Sino-Tibetan languages, linguistics preferred that its expansion is associated with the development of the Yangshao and/or Majiayao Neolithic cultures in China [12,25]. For Bantu languages, their dispersal is regarded as accompanied by the expansion of farmers from the tropical West African agricultural homeland in Nigeria and Cameroon [24,26]. For Arawak languages, their dispersal could be driven by the development of manioc cultivation in the ancient agricultural homeland in lowland South America [5]. However, the true associations among the demic diffusion, Neolithic/agricultural culture spread, and language dispersal remain controversial and require further detailed investigation.

1.2 Indo-European languages

The Indo-European languages are the mother tongue for the vast majority of populations residing in Europe, the Iranian plateau, and the northern Indian subcontinent [2]. This language family covers eight groups of Indo-European languages today which are Albanian, Armenian, Balto-Slavic, Celtic, Germanic, Hellenic, Indo-Iranian, and Italic. It is generally accepted that Indo-European languages all diverged from a single Proto-Indo-European language spoken approximately sometime in the Neolithic to the Early Bronze Age [24]. However, the concrete origin time and homeland of the Indo-European languages, known as the hottest debate in historical linguistics, have been the object of many competing hypotheses [2,24]. The major difficulty in solving the debate is the significant extinctions of Indo-European languages in history caused by the expansions of a few dominant subgroups including Germanic, Romance, Slavic, and Indo-Iranian [24].

Accordingly, there raised two hypotheses of the “Steppe hypothesis” and the “Anatolian hypothesis” [2,24,27]. The “Steppe hypothesis” proposes the homeland of Indo-European languages situated in the Pontic steppe region which lies to the north of the Caspian Sea. This hypothesis is also supported by the archaeological evidence [28] that the expansion of Indo-European languages could be attributed to the Kurgan seminomadic pastoralists who initiated the expansion roughly 5,000 to 6,000 years ago [28-31]. However, the objections state that horse domestication and riding had not appeared in this period which makes it implausible for pastoralists to transport their language to Western Europe far beyond the steppes [32,33]. In contrast to the “Steppe hypothesis”, the “Anatolian hypothesis” supported by the recent phylo-linguistic evidence posits that the Indo-European languages could originate in the Anatolia of the Crescent Fertile with the expansion accompanied by the spread of agriculture from Anatolia, beginning 8,000 to 9,000 years ago [33-36]. However, their objections include that the reconstructed Proto-Indo-European language lexicons show more associations with domesticated animals rather than crops [37,38]. Its close association with wheels and wheeled vehicles suggests the Indo-European language originated around 6,000 years ago during the invention of the wheels [39].

1.3 Sino-Tibetan languages

The Sino-Tibetan language family is distributed in a wide geographic range from East Asia, peninsular Southeast Asia, and the northern part of South Asia [40]. This language family is linguistically classified into about 40 well-established subgroups, of which those with the most speaker population size are: Sinitic (Chinese), Lolo-Burmese, and Tibetan. Although the reconstruction of the Sino-Tibetan language family at several low-level subgroups has been verified, their higher-level relationships remain unclear. The primary ongoing controversy is the classification or position of the Sinitic within the Sino-Tibetan language family. Accordingly, there are primary three hypotheses raised for the classification of the Sinitic. The first one proposes the dichotomic structure of the Sinitic and Tibeto-Burman languages that one branch of the Sino-Tibetan languages leads to the Sinitic and another one leads to the Tibeto-Burman languages [41-44]. Opposite to the first, the second considers the Sinitic as the lower-level subgroups of the Tibetan [45-47]. The third posits the

basal topology of Sino-Tibetan languages as a rake that Sinitic is one of several parallel clades [46-49].

Besides the classification of the Sinitic, the origin time and homeland of the Sino-Tibetan language family are also ongoing debates. These debates can be grouped into two competing hypotheses: The northern-origin hypothesis and the southwestern-origin hypothesis. As the most commonly cited one, the northern-origin hypothesis posits that the Sino-Tibetan language family originated in the upper and/or middle Yellow River Basin associating with the Neolith Yangshao (around 7,000-5,000 years BP) and/or Majiayao (5,500-4,000 years BP) culture, with an expansion driven by millet agriculture [40,44,50]. The southern-origin hypothesis posits the origin and expansion of the Sino-Tibetan language family occurred at approximately 9,000–10,000 years BP from the southwest region of East Asia. This hypothesis has two forms: the Sichuan-origin hypothesis and the Indian-origin hypothesis. The Sichuan-origin hypothesis proposes that the homeland of the Sino-Tibetan language family was located in the Sichuan Basin before 9,000 years BP with various outward migrations over time [51,52]. One group of languages traveled into northeast India and the other group of languages traveled northward into the Yellow River Basins becoming predecessors of Sinitic and Tibetic [51-53]. The Indian-origin hypothesis argues that the earliest speakers of Sino-Tibetan seemed to be highly diverse foragers rather than farmers in the eastern foothills of the Himalayas in Northeast India which is the area of the greatest linguistic diversity, around 9,000 years BP [46].

1.4 Bantu languages

Bantu languages are the largest branch of the Southern Bantoid languages within the Niger-Congo language family. These languages are widely spoken in the southeast of Cameroon, across Central Africa, Southeast Africa, and Southern Africa [24]. Although it is generally accepted that the Bantu language descended from a common Proto-Bantu language spoken in what is now eastern Nigeria and western Cameroon, whether the evolutionary history of Bantu languages is tree-like remains controversial [24,26]. While the tree model is favored by most researchers, objections have been raised by others. One major objection concentrates on the widespread lexical diffusion between different Bantu communities which makes the Bantu language evolution better represented as a dialectal chain rather than a tree [26].

It is acknowledged that the speakers of the Proto-Bantu language initiated a series of migrations out of the tropical West African agricultural homeland in eastern Nigeria and western Cameroon carrying agriculture with them for around 5,000 years BP [3,24]. This Bantu expansion came to dominate Sub-Saharan Africa east of Cameroon where Bantu peoples now constitute nearly the entire population [54,55]. It is one of the most influential and widespread cultural events that substitutes the life of indigenous forest foragers with a new and more sedentary way across a wide geographic area [24,56,57]. There are two possible dispersal routes of Bantu languages shaped by the two major events in the recent paleoenvironment history of Central Africa, respectively [3]. The first was a contraction of the Congo rainforest at its periphery along the coasts of southern Cameroon, Gabon, and Congo at around 4,000 years BP [58-60] which facilitates the Bantu population traveling

through the rainforest. The other was the emergence of patches of more or less open forests and wooded or grassland savannahs of the western part of the Congo Basin [61,62]. Finally, it facilitated the formation of a corridor known as the "Sangha River Interval" which could provide convenience to the north-south spread of certain typical savannah plant and animal species [59,63-65]. Accordingly, it may also have been a crucial route for the initial expansion of the Bantu population across the Equator.

1.5 Arawak languages

Arawak languages are a language family geographically distributed across lowland South America extending from Argentina to the Bahamas, and from the Amazon River to the Andes [5]. The homeland of Arawak languages is suggested to be situated in the region between the Rio Negro and the Orinoco because this region has the highest number of recorded Arawak languages [66]. However, the more precise location of the Arawak homeland remains in dispute. The deep clades of the Arawak language tree suggest four potential homelands: Western Amazonian, Atlantic seaboard, Central Amazon, and Northwest Amazonia [5]. The Western Amazonian origin is more likely than the last three because the divergence time of Arawak languages located in these homelands is much later than the divergence time of the Arawak languages in Western Amazonian [5]. It suggests that the Arawak languages could originate in the regions of the Purus River basin and the Andean foothills with a vast spread across lowland South America. This Arawak expansion is probably associated with the development of manioc cultivation in the transitional forests in the ecotone between southern lowland Amazonian rainforests, the Bolivian savannahs, and the dry forests of Central Brazil [67,68].

Supplementary Section 2: The Age-Area Hypothesis for diversity approach

Given that languages are the carriers of cultures, the common threads and challenges running through analyses of cultural spreads are to understand the spatial evolution of languages [69]. Using a variety of computational approaches such as the diversity hotspot approach (i.e., diversity approach) and Bayesian phylogeographic approach [70,71], many efforts on interdisciplinary alignment have been devoted to understanding the origins and dispersals of worldwide languages [2-5]. As the most popular phylogeny-based approach, the phylogeographic approaches, and their extensions infer the homelands of languages by modeling the changes in their geographic locations using a continuous-time model of drift [71]. In contrast, the diversity approach is a well-known phylogeny-free one with the heuristic algorithm which has been proposed based on the empirical intuition derived from the Age-Area Hypothesis (AAH) [69,70]. AAH, also known as centre-of-gravity or Sapir's principle [72], suggests that the language homeland should be located in the area with the largest language diversity [18,20].

However, the AAH has been long criticized for lacking theoretical underpinnings [18,69]. To be specific, there are no intrinsic associations between language diversification and homeland. For example, the homeland of languages can exhibit low diversity due to the migration of the native speakers out of their homeland such

as the origin of Semitic languages [18,73]. In addition, the mechanisms of language diversification are subject to several social factors that can also alter the language diversity of the homeland, such as cultural shifts and language replacement [74-78]. For example, cultural invasion may greatly reduce the language diversity of the homeland by the replacement of original languages with a few foreign languages.

Supplementary Section 3: The motivations and discussions for proposing the LVF

3.1 The limitations of the phylogeography

Language dispersal is usually inferred by the phylogeographic approach. The coordinate of language in the phylogeographic approaches is regarded as a discrete or continuous trait, whose evolution follows the drift model (also known as the random walk model) on a given language phylogenetic tree [2,3]. Such phylogenetic tree-based approaches have the limitation of capturing the horizontal transmissions among languages (e.g., borrowing and area diffusion) [79]. Although the structure of the phylogenetic tree is robust to the reasonable levels of horizontal transmissions [80], substantial horizontal transmissions in some of the language families and groups have made their evolution more consistent with the dialect chain or the rake-like topology rather than tree topology. Some prominent examples include the Oceanic languages across the Pacific settlements [79,81], Indo-Aryan languages across large parts of India [82], varieties of Arabic across north Africa and southwest Asia [83], the Turkic languages [84], the Chinese languages or dialects [85], and subgroups of the Romance [86], Germanic and Slavic families in Europe [87,88]. The evolutions of these language families and groups which have apparently violated the tree model cannot be modeled by the phylogenetic tree-based approaches. Therefore, the dependence on the phylogenetic tree actually limits the application of the phylogeographic approaches to more diverse language families and groups. On these grounds, it is important and meaningful to develop a more generalized approach without relying on the phylogenetic tree for inferring the dispersal patterns of the languages worldwide.

3.2 The motivations for proposing the LVF

Recent methodological advances in velocity field estimation provide an opportunity to infer the language dispersal patterns without relying on the phylogenetic tree. A velocity field can be visualized as a collection of arrows with given magnitudes and directions which are determined by a specific dynamic model [89]. It is widely utilized to visualize the speeds and directions of dynamic changes in natural systems like fluid movements (e.g., gases and liquids) in Physics [90] and cell differentiations in Biology [9]. The directions of velocity vectors within the velocity field thus compose a set of continuous trajectories of the dynamic changes of the natural system. Given its advantages in inferring the dynamic trajectory of the natural system, the application of the velocity field has been recently extended into the social sciences. Accordingly, it has become a powerful tool for inferring the dynamic trajectories of social systems such as demic diffusions [91] (e.g., human mobility), and cultural spreads [92] (e.g., Neolithic culture propagation). Note that humans are the carriers of

languages which are also the carriers of cultures. Accordingly, the dispersals of languages should share similar patterns with the demic diffusions and cultural spreads, which could also be modeled by the velocity field. It thus inspires us to propose the language velocity field estimation (LVF) to infer the language dispersal worldwide.

3.3 The advantages of the LVF

There are several advantages of the language velocity field estimation (LVF) compared to the phylogeographic approaches. The first one is the flexibility of the input data. The LVF does not require the reconstruction of the phylogenetic tree of languages but relies on the velocity field to determine from where the languages diffused to their current locations. Accordingly, the LVF can be applied to more diverse linguistic data, not only limited to lexical cognate data. For example, it can be used to infer the dispersal patterns of dialects using structural data (e.g., grammatical or phonological data). The second is the complete consideration of both vertical and horizontal transmissions in languages. To be specific, the dynamic model utilized in this study shares the same assumption about the language vertical transmission with the widely-used covarion model [93,94]. Moreover, it also considers the horizontal transmissions among different languages in reconstructing the language dispersal. The third one is the quantitative measurement of the dispersal directions of languages. To be specific, the direction of each arrow in the velocity field depicts the dispersal direction of the language located in that position. The fourth one is the universality of the application scenarios. Although the LVF treats the dispersal of languages like the fluids moving in the container, it also shows a good performance in the scenarios when the languages disperse as a random walk according to the simulated validations. Therefore, we hope that the language velocity estimation can greatly aid the spatial analysis of language evolution when the phylogeographic approaches are inapplicable.

3.4 The prospects of the LVF

According to the above-mentioned advantages, the language velocity field estimation (LVF) holds significant potential and promising development prospects. The first is the potential to facilitate interdisciplinary alignment from the spatial perspective. Unrestricted to the phylogenetic tree, the computational framework of LVF is also applicable to the genetic and archaeological data. To evaluate the consistency among interdisciplinary evidence when unraveling human prehistoric activities, numerous statistical methods and quantitative metrics can be designed and proposed based on the velocity field estimation. For instance, numerous metrics related to the similarity among the interdisciplinary velocity fields (e.g., linguistic, genetic, and archaeological velocity fields) can be devised to gauge the consistency among the human activity patterns drawn from interdisciplinary evidence. The second is the adaptability of the computational framework of LVF to a broad range of application scenarios. To be specific, the methods incorporated in LVF can be flexibly substituted to suit different practical situations. For example, (i) to reconstruct more intricated dispersal scenarios, we can employ more complicated dynamic models or machine

learning approaches to estimate the velocity field; (ii) to incorporate the various types of linguistic data especially language distant data [95], we can utilize other dimensional-reduction techniques such the Principle Coordinates Analysis (PCoA) [96] and Kernel Principle Component Analysis (KPCA) [97] to visualize the linguistic relatedness among language samples.

Nevertheless, we anticipate that some aspects of LVF can be improved in our future studies. Firstly, it is crucial to enhance the capability of LVF to estimate the time period of language dispersal. The LVF is a kind of pseudo-time analysis approach that can only estimate the language dispersal patterns from the spatial perspective. Since multiple dispersal centres may have been formed during the language dispersal history, it is difficult to distinguish the language homeland from these dispersal centres due to the lack of the language origin time. Accordingly, the improvement in the estimation of the language dispersal time period contributes to reconstructing a more complete and accurate language dispersal history. Secondly, it is critical to improving the ability of LVF to identify the language dispersal centre in more diverse dispersal scenarios. Since the native speakers could migrate out of their homeland [18,73], the language dispersal centre could be situated outside the geographic range of current language speakers. Under this circumstance, due to the lack of language samples in their homeland, the LVF can only estimate a secondary dispersal centre which was formed after the language diffused into their current geographic range. For example, the Oceanic languages are a branch of the Austronesian languages, whose dispersal route should be a part of the dispersal route of Austronesian languages from Southeast Asia across the Pacific settlements. Due to the lack of Oceanic language samples in Taiwan, China, the inferred dispersal centre of Oceanic languages is hard to be situated in the homeland of the Austronesian languages in Taiwan, China. Therefore, enhancing the ability to identify language dispersal centres located outside the current geographic range of language samples can significantly broaden the potential applications of LVF.

Supplementary Methods

Supplementary Section 1: Mathematical formulas and derivations for the LVF

1.1 The calculation of the velocity field

1.1.1 Principle for the parametric estimation of prestige in the dynamic model

Before implementing our dynamic model for reconstructing the past state frequency of each linguistic trait, it is imperative to ascertain the prestige parameter within the dynamic model. Conventionally, such estimation is achieved by fitting the model against empirical time-series data gathered at different time points. However, in this particular study, the linguistic data are cross-sectional of which the language samples are regarded as being collected at the same time. Consequently, the key parameter of prestige in our model is unable to be estimated through conventional model-fitting techniques. It hence raises a significant challenge to estimate the key parameters based on the cross-sectional data.

It is noted that our dynamic model is adapted from the well-attested Abrams-Strogatz (AS) model [98] where the corresponding prestige parameter has been redefined as the language inheritance rate by the Zhang-Gong model [10]. The Zhang-Gong model underscores the remarkable parallels between linguistic inheritance and genetic inheritance. It posits that parents can transmit the languages they speak to their offspring. Languages with higher inheritance rates will be poised for prosperity in subsequent generations, whereas those with lower inheritance rates will gradually diminish in usage. Consequently, the prestige of state j for trait i (s_j^i, j = 0 or 1) in our dynamic model can be measured by its probability of producing offspring that remain in state j after a unit of time (one generation). In other words, the prestige of state j for trait i (s_j^i, j = 0 or 1) signifies the probability of trait i being in state j that remains in state j after a unit of time (one generation) (the definition of a unit of time can be found in Supplementary Notes section 1.2.1).

In practical terms, both languages and genes would typically undergo variations during the process of inheritance. To model genetic inheritance, Felsenstein introduced a statistical model built upon the Poisson process [15]. This model has been proven highly effective in explaining DNA substitutions in genetic inheritance. Given the notable resemblance between genetic and linguistic inheritance, we also conducted the Poisson process to model linguistic inheritance and compute the prestige parameter (the detailed explanation for adopting the Poisson process can be found in Supplementary Notes section 1.2.2). More specifically, we postulate that the offspring of trait i can inherit the state of trait i or undergo multiple transitions to another state. Such a transition takes place at a constant rate λ which is referred to as the mutation rate. Within a very short time interval Δt (considerably shorter than one generation), such a transition only can occur one time at

most. For instance, let's consider trait i currently in state 0. The offspring of trait i generated after time t may alter from state 0 to state 1 or remain in state 0. In other words, a trait i in state j can either persist in that state j or switch to another state after time t.

Let $N(t)$ be the number of occurrences of the state transition in trait i during time t. According to the Poisson process, the probability of n transitions occurring during time t is given by $P(N(t)=n)=e^{-\lambda t}\frac{(\lambda t)^n}{n!}$. Therefore, the probability that no state transition has occurred during time t is $P(N(t)=0)=e^{-\lambda t}$. The probability that at least one state transition has occurred during time t is $P(N(t)>0)=1-P(N(t)=0)=1-e^{-\lambda t}$. Accordingly, the probability of state u transiting to state j (where $u \neq j$) during time t (i.e., $p_{uj}(t)$) can be given by the product of the probability that state u has occurred at least one transition into any other states (i.e., $P(N(t)>0)$) and the probability of such transition that eventually leads back to state j (i.e., π_j). Mathematically, $p_{uj}(t)=P(N(t)>0)\pi_j=(1-e^{-\lambda t})\pi_j$. Likewise, the probability of state u transiting to state u during time t (i.e., $p_{uu}(t)$) can be calculated as the sum of the probability that no transition has occurred (i.e., $P(N(t)=0)$) and the probability that at least one transition has occurred but ends up with transition back to state u (i.e., $P(N(t)>0)\pi_u$). Mathematically, $p_{uu}(t)=P(N(t)=0)+P(N(t)>0)\pi_u=e^{-\lambda t}+(1-e^{-\lambda t})\pi_u$. Consequently, the probability of state u (u = 0 or 1) in trait i transiting to state j after time t can be calculated as Equation (S1).

$$P_{uj}^i(t)=e^{-\lambda t}\delta_{uj}+(1-e^{-\lambda t})\pi_j^i \tag{S1}$$

Here, $\delta_{uj}=1$, if $u=j$. $\delta_{uj}=0$, if $u\neq j$. π_j^i denotes the transition probability that a transition will result in any current state of trait i eventually being replaced with state j [15,99]. In this study, we set the transition probability π_j^i as the frequency of state j of trait i in the language samples. Since lacking temporal information regarding linguistic traits, we consider that based on this parametric setting, the Poisson process can better interpret the formation of the observed state distribution in each linguistic trait.

Furthermore, in this study, we set $\lambda = 1$ which describes a most general scenario in which the state of each trait in a language can only change once on average in a unit of time (one generation). This setting has been justified by both simulated and empirical validations. Following the definition of the prestige parameter, the prestige of state 1 and state 0 of trait i (s_0^i and s_1^i) can be calculated using Equation (S1) with the setting of t = 1 (one generation) as shown in Equation (S2).

$$\begin{cases} s_1^i=P_{11}^i(1)=e^{-1}+(1-e^{-1})\pi_1^i \\ s_0^i=P_{00}^i(1)=e^{-1}+(1-e^{-1})\pi_0^i \end{cases} \tag{S2}$$

1.1.2 The reconstruction of the past states for linguistic traits using a dynamic model

The analytic method for reconstructing the state frequency of a linguistic trait. We here illustrate how to reconstruct the past state frequency for each linguistic trait based on its current state frequency using our dynamic model. It is noted that our model as shown in Equation (S3) can be rewritten as Equation (S4). By solving Equation (S4), we can derive the past state frequency of trait *i*. To be specific, we set the current time as $t = t_0$. By integrating Equation (S4) from t_0 to t_{-m} as Equation (S5), we can calculate the state frequency of trait *i* at t_{-m} before time t_0 ($t_{-m} < t_0$.) as Equation (S6).

$$\begin{cases} \frac{dx_0^i}{dt} = x_1^i q_{10}(x_0^i, s_0^i) - x_0^i q_{01}(x_1^i, s_1^i) \\ \frac{dx_1^i}{dt} = x_0^i q_{01}(x_1^i, s_1^i) - x_1^i q_{10}(x_0^i, s_0^i) \end{cases} \qquad \text{(S3)}$$

$$\frac{dx_1^i}{dt} = (s_1^i - s_0^i)(1 - x_1^i)x_1^i \qquad \text{(S4)}$$

$$\int_{x_1^i(t_0)}^{x_1^i(t_{-m})} \frac{1}{(1-x_1^i)x_1^i} dx_1^i = \int_{t_0}^{t_{-m}} (s_1^i - s_0^i) dt \qquad \text{(S5)}$$

$$x_1^i(t_{-m}) = \frac{1}{1+(\frac{1}{x_1^i(t_0)}-1)e^{-(s_1^i-s_0^i)(t_{-m}-t_0)}} \qquad \text{(S6)}$$

As we can see in Equation (S6), the value $x_1^i(t_0 - m)$ at $t_{-m} = t_0 - m$ remains constant for any settings of t_0, once the initial value $x_1^i(t_0)$ and the difference between t_0 and m (i.e., $t_0 - m$) stay constant. For convenience, we set $t_0 = 0$ in this study. Therefore, the state frequency of trait *i* at time $t_0 - m = -m < 0$ (m times before the current time $t_0 = 0$) can be calculated based on Equation (S6) as Equation (S7).

$$x_1^i(-m) = \frac{1}{1+(\frac{1}{x_1^i(0)}-1)e^{(s_1^i-s_0^i)m}} \qquad \text{(S7)}$$

The numerical method for reconstructing the state frequency of a linguistic trait. It is noted that our LVF approach allows different dynamic models for reconstructing past state frequencies of linguistic traits. However, an analytical solution like Equation (S6) cannot be obtained by all dynamic models. Therefore, numerical iterative methods such as the Runge-Kutta method [100] can be utilized to approximate the solutions for the dynamic model. However, these iterative methods are harnessed for model prediction rather than reconstruction which cannot be conducted directly in our approach. Noting these, we introduce a strategy that transforms the problem of the model reconstruction into model prediction. Subsequently, numerical analysis can be employed to approximate the model solutions.

To be specific, Equation (S4) can be expressed in a more general form as Equation (S8), where $F^i(x_1^i(t)) = (s_1^i - s_0^i)(1 - x_1^i(t))x_1^i(t)$ is a continuous function and x^i is the known state frequency of trait

i at time t_0. It is noted that $F^i(x_1^i(t))$ can be regarded as the composite of two functions: $F^i(u) = (s_1^i - s_0^i)(1-u)u$ and $u = x_1^i(t)$.

$$\begin{cases} \frac{dx_1^i(t)}{dt} = F^i(x_1^i(t)) \\ x_1^i(t_0) = x^i \end{cases} \tag{S8}$$

We search for a dynamic model whose solution ($y_1^i(t)$) is symmetric to the solution of Equation (S8) ($x_1^i(t)$) with respect to $t = t_0$ (i.e., $y_1^i(t_0+m) = x_1^i(t_0-m)$). Accordingly, the derivative of $y_1^i(t)$ can be solved following Equation (S9).

$$\frac{dy_1^i(t)}{dt} = \lim_{dt\to 0} \frac{y_1^i(t+dt)-y_1^i(t)}{dt}$$

$$= \lim_{dt\to 0} \frac{y_1^i(t_0-t_0+t+dt)-y_1^i(t_0-t_0+t)}{dt}$$

$$= \lim_{dt\to 0} \frac{x_1^i(t_0+t_0-t-dt)-x_1^i(t_0+t_0-t)}{dt}$$

$$= \lim_{dt\to 0} -\frac{x_1^i(t_0+t_0-t-dt)-x_1^i(t_0+t_0-t)}{-dt}$$

$$= \lim_{dt\to 0} -\frac{x_1^i(t_0+t_0-t+\Delta t)-x_1^i(t_0+t_0-t)}{\Delta t} \quad (\Delta t = -dt)$$

$$= \lim_{\Delta t\to 0} -\frac{x_1^i(t_0+t_0-t+\Delta t)-x_1^i(t_0+t_0-t)}{\Delta t}$$

$$= -F^i(x_1^i(t_0+t_0-t))$$

$$= -F^i(y_1^i(t)) \tag{S9}$$

According to Equation (S9), The dynamic model which has the solution $y_1^i(t)$ symmetric to the solution $x_1^i(t)$ with respect to $t = t_0$ can thus be derived as Equation (S10).

$$\begin{cases} \frac{dy_1^i(t)}{dt} = -F(y_1^i(t)) \\ y_1^i(t_0) = x^i \end{cases} \tag{S10}$$

Therefore, the reconstruction of the state frequency of linguistic trait i at time $t_{-m} = -m < 0$ before current time $t_0 = 0$ based on Equation (S8) is equal to the prediction of the state frequency of linguistic trait i at time $t_m = m > 0$ after current time $t_0 = 0$ based on Equation (S10) (i.e., $y_1^i(m) = x_1^i(-m)$). And, $y_1^i(m)$ can be

calculated by applying the Runge-Kutta methods to Equation (S10). The Runge-Kutta methods can be implemented by package '*deSolve*' [101] in R (4.3.1).

In comparison to the analytic method, the numerical method is more general but less efficient in operation. In practice, we find that the average difference between the past state frequencies calculated by the analytic method and the numerical method is smaller than 1×10^{-6}. However, given the operation efficiency of our LVF approach, we employ the analytical method in this study for reconstructing the past state frequencies of linguistic traits of a language.

1.1.3 Velocity field establishment

Based on the computational procedures illustrated in Supplementary Methods section 1.1.2, we have the capability to reconstruct the state frequency of trait i for language l at time $t_{-m} = -m < 0$ (${}^{l}x_1^{i}(-m)$) before the current time $t_0 = 0$. In practice, for each linguistic trait, we reconstruct its past state frequency ${}^{l}x_1^{i}(-m)$, i = 1, 2, …, p. Therefore, the velocity vector of this language l is a p-dimensional vector which can be calculated as Equation (S11).

$$\mathbf{V}_l = \frac{\mathbf{X}_l(0)-\mathbf{X}_l(-m)}{m} \tag{S11}$$

Here $\mathbf{X}_l(0) = [{}^{l}x_1^{1}(0), {}^{l}x_1^{2}(0), .., {}^{l}x_1^{p}(0)]^T$ represents the state frequencies of p linguistic traits for language l at current time $t_0 = 0$. ${}^{l}x_1^{i}(0)$ signifies the state frequency of trait i (i = 1, 2, …, p) for language l at current time t_0 = 0. Similarly, $\mathbf{X}_l(-m) = [{}^{l}x_1^{1}(-m), {}^{l}x_1^{2}(-m), .., {}^{l}x_1^{p}(-m)]^T$ signifies the state frequencies of p linguistic traits for language l at past time $t_{-m} = -m$. ${}^{l}x_1^{i}(-m)$ denotes the state frequency of trait i (i = 1, 2, …, p) for language l at past time $t_{-m} = -m$. Therefore, $\mathbf{V}_l$ measures the changes in state frequencies of linguistic traits for language l in a unit of time. For each language family or group, we can calculate the velocity vectors for its n language samples, hence composing a velocity field that can be expressed as a velocity matrix ($\mathbf{V}$) as Equation (S12).

$$\mathbf{V} = \frac{(\mathbf{X}_{current}(0)-\mathbf{X}_{past}(-m))}{m} = \begin{bmatrix} \mathbf{V}_1^T \\ \mathbf{V}_2^T \\ \vdots \\ \mathbf{V}_n^T \end{bmatrix} \tag{S12}$$

Here, the matrix $\mathbf{X}_{current}(0) = \begin{bmatrix} \mathbf{X}_1^T(0) \\ \mathbf{X}_2^T(0) \\ \vdots \\ \mathbf{X}_n^T(0) \end{bmatrix}$ denotes the state frequencies of linguistic traits for n language

samples at the current time. The matrix $\mathbf{X}_{past}(-m) = \begin{bmatrix} \mathbf{X}_1^T(-m) \\ \mathbf{X}_2^T(-m) \\ \vdots \\ \mathbf{X}_n^T(-m) \end{bmatrix}$ signifies the state frequencies of linguistic traits for n language samples at past time $t_{-m} = -m$. In this study, we set $m = 1$, which has been verified in both simulated and empirical validations.

1.2 The projection of the velocity field

1.2.1 PCA projection of the velocity field

To measure the linguistic relatedness among the language samples, we perform the principal component analysis (PCA) to extract two optimal principal components (PC1 and PC2) noted as $\mathbf{D}^{PC}$ from an $n \times p$ matrix $\mathbf{D}$. This matrix $\mathbf{D}$ contains n language samples and p binary-coded linguistic traits. According to the computational procedure of PCA, we first estimate an orthogonal transformation **A,** which is an orthogonal matrix containing the eigenvectors of the covariance matrix of $\mathbf{D}$. Subsequently, $\mathbf{D}^{PC}$ can be computed by the matrix product of $\mathbf{D}$ and the first two columns of $\mathbf{A}$ noted as $\mathbf{A}_2$ following Equation (S13).

$$\mathbf{D}^{PC} = \begin{bmatrix} (\mathbf{D}_1^{PC})^T \\ \vdots \\ (\mathbf{D}_n^{PC})^T \end{bmatrix} = \mathbf{D}\mathbf{A}_2 \tag{S13}$$

Here, $\mathbf{A}_2$ is a $n \times 2$ matrix that contains the first two columns of the orthogonal matrix $\mathbf{A}$. $\mathbf{D}_l^{PC}(l = 1,2, \dots n)$ signifies the PC values of language l. It is noted that $\mathbf{D}^{PC}$ can be regarded as the linear projection of $\mathbf{D}$ within the 2-dimensional PC space, which is accomplished through a linear transformation facilitated by $\mathbf{A}_2$. Utilizing $\mathbf{A}_2$, we can further project the velocity field $\mathbf{V}$ into the 2-dimensional PC space formed by the aforementioned two principal components, to derive a two-dimensional velocity field denoted as $\mathbf{V}^{PC}$ following Equation (S14) (Fig. 1e1).

$$\mathbf{V}^{PC} = \begin{bmatrix} (\mathbf{V}_1^{PC})^T \\ \vdots \\ (\mathbf{V}_n^{PC})^T \end{bmatrix} = \mathbf{V}\mathbf{A}_2 \tag{S14}$$

1.2.2 Kernel projection of the velocity field

Through PCA projection, we have projected the p-dimensional velocity field $\mathbf{V}$ into the 2-dimensional PC space. This 2-dimensional velocity field $\mathbf{V}^{PC}$ illustrates the diachronic evolutionary trajectories of linguistic traits that shape the observed linguistic relatedness. By projecting the $\mathbf{V}^{PC}$ into the geographic space based on the observed correlation between linguistic relatedness and language geography (Fig. 1f1), we can derive a velocity field within the geographic space ($\mathbf{V}^{Geo}$) that outlines the language dispersal trajectories (Fig. 1f2).

Here, we utilize and modify the kernel projection proposed by La Manno et al. [9] to accomplish this geographic projection of the velocity field $\mathbf{V}^{PC}$. The rationale of the kernel projection is to search for the velocity vector of each language sample in the geographic space, ensuring that its correlation with the language distribution in the PC space can be best preserved within the geographic space (Fig. 1f1). In other words, this kernel projection can be regarded as transforming the evolutionary directions of linguistic traits into language dispersal directions according to the observed correlation between linguistic relatedness and language geography (Fig. 1f).

It is noted that the language samples situated in close geographic proximity may share more similarities in their traits thereby displaying a closer distribution in the PC space. However, when language samples are situated far apart from each other in the PC space, their linguistic relatedness may not be correlated well with their language geography. Therefore, incorporating language samples that are far from a specific language sample in the PC space for the kernel projection of its velocity vector would largely diminish the correlation between their linguistic relatedness and language geography. It would thus introduce more uncertainties into the estimation of the velocity vector of this language sample in the geographic space. On the contrary, selecting too less language samples for kernel projection would impede the accurate assessment of the correlation between linguistic relatedness and language geography. Therefore, it is essential to consider both the distances among language samples in PC space and the number of language samples utilized for kernel projection simultaneously when estimating the velocity vectors in geographic space. Noting these, we modify the original kernel projection method to employ the so-called "local kernel projection" that accounts for the optimal number of language samples as well as their distances within PC space when projecting the velocity vectors into geographic space.

The velocity vectors in geographic space are estimated based on two kernels: Exponential and Gaussian kernels. To be specific, we first calculate a transition probability matrix or correlation matrix $\mathbf{P}=[P_{lj}]_{l=1, 2, ..., n; j=1, 2, ..., n}$. This transition probability matrix $\mathbf{P}$ quantifies the correlation between velocity vectors and the language distribution within PC space. Second, we estimate the velocity vectors in the geographic space utilizing this matrix $\mathbf{P}$ as weights based on the correlation between linguistic relatedness and language geography.

Estimating transition probability matrix. Each P_{lj} is utilized as a weight to compute the velocity vector of language l in geographic space, which is estimated through the product of the two components as shown in Equation (S15). In short, the first component is the exponential kernel transformation of the cosine similarity between the velocity vector of language l ($\mathbf{V}_l^{PC}$) and its synchronic distinction from any language j ($\mathbf{r}_{lj}^{PC}$) within PC space. The second component is the Gaussian kernel transformation of the magnitude of this synchronic distinction ($\|\mathbf{r}_{lj}^{PC}\|$) within PC space.

$$P_{lj} = exp(\frac{cos(q(\mathbf{r}_{lj}^{PC}), q(\mathbf{V}_l^{PC}))}{\sigma_1}) \, exp(-\frac{\left\| q(\mathbf{r}_{lj}^{PC}) \right\|^2}{2\sigma_2^2})$$

$$= exp(\frac{cos(q(\mathbf{r}_{lj}^{PC}), q(\mathbf{V}_l^{PC}))}{\sigma_1} - \frac{\left\| q\left(\mathbf{r}_{lj}^{PC}\right) \right\|^2}{2\sigma_2^2}) \tag{S15}$$

$$\mathbf{r}_{lj}^{PC} = \mathbf{D}_j^{PC} - \mathbf{D}_l^{PC} \tag{S16}$$

$$cos(\mathbf{x}, \mathbf{y}) = \frac{\mathbf{x}^T \mathbf{y}}{\|\mathbf{x}\| \|\mathbf{y}\|} \tag{S17}$$

$$q(\mathbf{x}) = sgn(\mathbf{x}) \sqrt{|\mathbf{x}|} \tag{S18}$$

Here, σ_1 and σ_2 are the bandwidths of Exponential and Gaussian kernels. In this study, we recommend that these two bandwidths could be set as 0.1, 0.5, 1, 5, 10, or 15. Particularly, the σ_2 governs the extent of scaling for the similarity/correlation between the $\mathbf{V}_l^{PC}$ and $\mathbf{r}_{lj}^{PC}$. σ_2 is suggested to be set as a smaller value when the language dispersal is more largely constrained by ecological or environmental factors such as oceans, islands, mountains, and plateaus. This is because those constraints would lead to the inconsistency between linguistic relatedness and language geography, such as languages sharing more similarities but being geographically distant from each other. $q(\mathbf{r}_{lj}^{PC})$ signifies the synchronic change from languages l to j within PC space transformed with a variance-stabilizing (elementwise) transformation q [9]. Similarly, $q(\mathbf{V}_l^{PC})$ is the q-transformed velocity vector of language l within PC space as shown in Equation (S16-S18).

Specifically, the cosine similarity between $\mathbf{V}_l^{PC}$ and $\mathbf{r}_{lj}^{PC}$ quantifies the similarity/correlation between the diachronic position change of language l and the synchronic position change from languages l to j within PC space. This measures the similarity/correlation between the velocity vector of language l and the distribution of languages l and j within PC space. When $\mathbf{V}_l^{PC}$ and $\mathbf{r}_{lj}^{PC}$ share a higher similarity/correlation, the value of the Exponential kernel would be larger. However, we consider that when two languages l and j are distributed far from each other within PC space, the similarity/correlation between $\mathbf{V}_l^{PC}$ and $\mathbf{r}_{lj}^{PC}$ would be harder to be retained within geographic space, due to the less consistency between the linguistic relatedness of language l and j and their geographic proximity. Therefore, we introduce the Gaussian kernel to adjust the similarity/correlation between $\mathbf{V}_l^{PC}$ and $\mathbf{r}_{lj}^{PC}$. When the Euclidean distance between languages l and j within PC space is larger, the value of the Gaussian kernel would be lower. In other words, if languages l and j exhibit a greater distance within PC space, the weight of language j in estimating the velocity vector of language l within geographic space would be smaller. This reduces the uncertainty in estimating the velocity vector of language l in geographic space, raised by the inconsistency between the linguistic relatedness of languages l and j and their geographic proximity. Therefore, P_{lj} is determined by the similarity/correlation between $\mathbf{V}_l^{PC}$ and $\mathbf{r}_{lj}^{PC}$ which is further scaled based on the $\left\|\mathbf{r}_{lj}^{PC}\right\|$ within PC space.

Estimating velocity vectors within geographic space. To determine the optimal number (s) of language samples utilized for the kernel projection of velocity vectors, we cluster the language samples within the PC space based on the metric of optimum average silhouette width [102]. Specifically, the value of s is calculated as $s = n/n.clust$ where *n.clust* is the optimal cluster size of the language samples within PC space calculated based on the optimum average silhouette width [102]. In this study, *n.clust* is estimated using the "*pamk*" function of the "*fpc*" package [103] in R (4.3.1). Subsequently, we normalize the transition matrix **P** by rows following the work of La Manno et al. [9] so that each P_{lj} can be regarded as a correlation coefficient or a weight ranging from 0 to 1 and satisfies $\sum_{j=1}^{n} P_{lj} = 1$. Given the coordinates of the longitudes and latitudes of the language samples surrounding language l, the velocity vector of language l in the geographic space is ultimately estimated as Equation (S19). As we can see in Equation (S19), each velocity vector within geographic space can be regarded as being estimated by weighting the synchronic differences among geographic coordinates of language samples by the matrix **P**.

$$\mathbf{V}_{\boldsymbol{l}}^{\boldsymbol{Geo}} = \sum_{j=1}^{s} P_{lj} \frac{(\mathbf{C}_j - \mathbf{C}_l)}{\|\mathbf{C}_j - \mathbf{C}_l\|} - \frac{1}{s} \sum_{j=1}^{s} \frac{(\mathbf{C}_j - \mathbf{C}_l)}{\|\mathbf{C}_j - \mathbf{C}_l\|}$$

$$= \sum_{j=1}^{s} (P_{lj} - \frac{1}{s}) \frac{(\mathbf{C}_j - \mathbf{C}_l)}{\|\mathbf{C}_j - \mathbf{C}_l\|} \qquad \text{(S19)}$$

Here, subtracting $1/s$ corrects the estimation for the non-uniform density of language samples within geographic space [9]. $\mathbf{C}_l$ denotes the coordinate of longitude and latitude of language l. $\mathbf{C}_j$ signifies the coordinate of longitude and latitude of language j which is one of the s closest language samples to language l within the PC space. The velocity field within the geographic space is noted as the matrix $\mathbf{V}^{\boldsymbol{Geo}} = [\mathbf{V}_{\mathbf{1}}^{\boldsymbol{Geo}}, \ldots, \mathbf{V}_{\boldsymbol{n}}^{\boldsymbol{Geo}}]^T$.

1.2.3 Spatial smoothing for the velocity field

To better visualize the velocity field in the geographic space, we implement the spatial smoothing approach on $\mathbf{V}^{Geo}$. To be specific, we first scale the magnitude/length of the velocity vector for each language sample within the geographic space ($\|\mathbf{V}_{\boldsymbol{l}}^{\boldsymbol{Geo}}\|$) to match its magnitude/length within the PC space ($\|\mathbf{V}_{\boldsymbol{l}}^{\boldsymbol{PC}}\|$) based on Equation (S20) (Fig. 1f2).

$$\mathbf{V}_{\boldsymbol{l}}^{\boldsymbol{Geo-scale}} = \frac{\mathbf{V}_{\boldsymbol{l}}^{\boldsymbol{Geo}}}{\|\mathbf{V}_{\boldsymbol{l}}^{\boldsymbol{Geo}}\|} \|\mathbf{V}_{\boldsymbol{l}}^{\boldsymbol{PC}}\| \qquad \text{(S20)}$$

Subsequently, we apply the Gaussian kernel smoothing to adjust the magnitude/length of the velocity vector within geographic space as Equation (S21).

$$\mathbf{V}_{\boldsymbol{l}}^{\boldsymbol{Geo-scale-smooth}} = \frac{\mathbf{V}_{\boldsymbol{l}}^{\boldsymbol{Geo-scale}}}{\|\mathbf{V}_{\boldsymbol{l}}^{\boldsymbol{Geo-scale}}\|} \sum_{j=1}^{n} K_\sigma(\mathbf{C}_{\boldsymbol{l}}, \mathbf{C}_{\boldsymbol{j}}) ||\mathbf{V}_{\boldsymbol{j}}^{\boldsymbol{Geo-scale}}|| \qquad \text{(S21)}$$

The kernel function K_σ for two vectors **x** and **y** is defined as Equation (S22) in which the σ is the bandwidth

of the Gaussian kernel.

$$K_\sigma(\mathbf{x}, \mathbf{y}) = exp(\frac{-\|\mathbf{x}-\mathbf{y}\|^2}{2\sigma^2}) \tag{S22}$$

1.2.4 Grid smoothing for the velocity field

Visualization of velocity vectors is not practical for language samples that manifest very dense or sparse distributions. For such cases, we further visualize a velocity field ($\mathbf{V}^{Grid}$) showing local group velocity vectors evaluated on regular grid points (hereafter grid-smoothed velocity field). Each grid-smoothed velocity vector ($\mathbf{V}_g^{Grid}$) is constructed by applying Gaussian kernel smoothing to the velocity vectors of language samples which are geographically near to each grid point as Equation (S23) (Fig. 1g).

$$\mathbf{V}_g^{Grid} = \sum_l K_\sigma(\mathbf{C}_g^{Grid}, \mathbf{C}_l)\mathbf{V}_l^{Geo-scale-smooth} \tag{S23}$$

Here, $\mathbf{V}_l^{Geo\text{-}scale\text{-}smooth}$ is the velocity vector of language l in geographic space that has been spatial-smoothed. $\mathbf{C}_g^{Grid} = [lon.grid, lat.grid]^T$ denotes the geographic coordinate of longitude and latitude of grid g. $\mathbf{C}_l$ is the geographic coordinate of the longitude and latitude of language l which is located in the cover $[lon.grid\text{-}b, lon.grid\text{+}b] \times [lat.grid\text{-}b, lat.grid\text{+}b]$. b denotes the bandwidth of this cover. The kernel function K_σ is the same as shown in Equation (S21). La Manno et al. [9] suggest that the size/number of the grid points should be chosen specifically depending on the visual scale of the figure.

1.3 Language dispersal centre inference

1.3.1 Inferring the language dispersal centre

To infer the language dispersal centre, we design two different strategies namely radiative and chained strategies rooted on the grid-smoothed velocity field in the geographic space (detailed explanations of the dispersal centre inference can be found in Supplementary Notes section 1.3). It is noted that we only employ the radiative strategy in this study since it has been proven effective in both simulated and empirical applications. Nevertheless, the chained strategy is worth trying while the radiative strategy loses its efficiency under the circumstance of language dispersal exhibiting a significant chained topology. In the chained topology, the velocity vectors may all point roughly in the same direction rather than point outwardly in all directions around the dispersal centre. Therefore, we also present the mathematical formulas for the chained strategy in this section, although this strategy may be not adequately compatible with the linguistic reality. In the future study, we look forward to improving the chained strategy for identifying the language dispersal centre.

For the radiative strategy, we first scale the grid-smoothed velocity vectors following Equation (S24). Secondly, we calculate the average for the variance of the grid-smoothed velocity vectors around each grid point in each dimension as Equation (S25). This average variance measures the degree of the outwards radiation pattern of the grid-smoothed velocity vectors around each grid point.

$$\mathbf{V}_g^{Grid-scale} = [x_g^{Grid-scale}, y_g^{Grid-scale}]^T = \frac{\mathbf{V}_g^{Grid}}{\|\mathbf{V}_g^{Grid}\|} \quad \text{(S24)}$$

$$\sigma_g^2 = \frac{1}{2(s-1)} \sum_{u=1}^{s} [(x_u^{Grid-scale} - \frac{1}{s}\sum_{u=1}^{s} x_u^{Grid-scale})^2 + (y_u^{Grid-scale} - \frac{1}{s}\sum_{u=1}^{s} y_u^{Grid-scale})^2] \quad \text{(S25)}$$

Here, $[x_u^{Grid-scale}]_{u=1}^{s}$ and $[y_u^{Grid-scale}]_{u=1}^{s}$ are the values of the grid-smoothed velocity vectors of s grid points that are nearest to the grid g (includes itself) in two dimensions. Accordingly, we assume that the potential dispersal centre should be situated in the geographic location of the grid point which has the largest average variance (σ^2) (Fig. 1g).

For the chained strategy, we first calculate the convex hull of the language samples according to their geographic coordinates. Secondly, we calculate the average grid-smoothed velocity vector ($\bar{\mathbf{V}}$) as Equation (S26).

$$\bar{\mathbf{V}} = \frac{1}{M} \sum_{g=1}^{M} \mathbf{V}_g^{Grid} \quad \text{(S26)}$$

Here, M is the number of grid points in the geographic space. This average grid-smoothed velocity vector $\bar{\mathbf{V}}$ reflects the average dispersal direction of the language samples. Subsequently, based on the cosine similarity as Equation (S27), we compute the correlation between the velocity vector of each language sample which is located on the border of the convex hull and this average grid-smoothed velocity vector $\bar{\mathbf{V}}$.

$$Cos_l = \frac{\bar{\mathbf{V}}^T \mathbf{V}_l^{Geo}}{\|\bar{\mathbf{V}}\| \|\mathbf{V}_l^{Geo}\|} \quad \text{(S27)}$$

The coordinate of the language sample that is located on the border of the convex hull and manifests the highest correlation with the $\bar{\mathbf{V}}$ is regarded as the dispersal centre.

1.3.2 Inferring the Standard Deviation of the language dispersal centre

To ascertain the geographic range of the dispersal centre, we perform the traditional jackknife resampling [19] to estimate the Standard Deviation (SD) values for the longitude and latitude of the language dispersal centre respectively (Supplementary Table 2). Furthermore, we observe that the SDs of the dispersal centres are quite different among the four language families and groups in terms of both longitudinal and latitudinal perspectives. We believe that such a difference could be attributed to the spatial dispersion of language samples

across the geographic space. To be specific, the greater spatial dispersion of language samples would introduce increased uncertainties into the SD value of the coordinate of the estimated language dispersal centre. The geographic dispersion of longitude or latitude is measured by Equation (S28).

$$Dispersion = \frac{\frac{\sum_{i=1}^{n-1}(x_{(i+1)}-x_{(i)})}{n}}{(x_{(n)}-x_{(1)})} \quad \text{(S28)}$$

Here, $x_{(i)}$ is the ith order statistic (i^{th} smallest value) of the longitudes or latitudes of the n language samples of a given language family or group. To examine the associations between the dispersion and SDs of longitude and latitude of the language dispersal centre, we further perform the linear regression analysis. The results show that there is a high association between the dispersion and the SD value in the longitudinal perspective (Longitude: Adj-R2 = 0.999, p-value = 3×10^{-4}; Latitude: Adj-R2 = 0.307, p-value = 0.6413) (Supplementary Fig. 11). It confirms our conjecture that the dispersal centre estimated by LVF could be influenced by the spatial heterogeneity of language samples distributed across the geographic space.

1.3.3 Comparison with the diversity approach

We conduct a comparison between LVF and the traditional diversity approach [20,40,104]. The diversity approach is also a phylogenetic tree-free approach that simply puts the dispersal centre in the area with the greatest linguistic diversity. To be specific, The diversity approach postulates that early divergence would manifest a higher diversification rate [70]. Such a higher diversification rate would subsequently lead to the rapid birth of numerous distinct languages around the dispersal centre [70]. According to this postulation, the language dispersal centre should be situated in the area encompassing the greatest linguistic diversity [20]. In other words, the languages situated around the dispersal centre should manifest the greatest dissimilarities in their linguistic traits. Therefore, languages located in the area with the largest diversity are expected to manifest the highest degree of uncertainty in possessing different trait states. Such uncertainty can be measured through the state frequencies of linguistic traits converted from their binary states. For instance, the higher state frequency of a trait indicates a greater probability of exhibiting state 1 but a lower probability of displaying state 0 within a specific geographic area. However, the trait with much higher or lower state frequency does not exhibit higher diversity. In contrast, when a trait exhibits a state frequency identical to 0.5, it signifies the highest degree of uncertainty regarding whether this trait would exhibit state 1 or state 0. Under this circumstance, this trait is regarded as exhibiting the greatest diversity.

The Information entropy is a widely used index to measure the degree of the uncertainty of a random variable [105]. The higher information entropy indicates a higher degree of diversity for a random variable. Accordingly, we adopt the information entropy to measure the diversity of each linguistic trait. The linguistic diversity of a language is calculated as the sum of the information entropy of each linguistic trait as Equation (S29).

$$Div_l = \sum_{i=1}^{p}[-{}^{l}x_1^i(0)\log\left({}^{l}x_1^i(0)\right) - (1-{}^{l}x_1^i(0))\log\left(1-{}^{l}x_1^i(0)\right)] \quad \text{(S29)}$$

Here, ${}^{l}x_1^i(0)$ signifies the state frequency of trait *i* (*i*=1, 2, …, *p*) in language *l* at the current time $t_0 = 0$. For each language family or group, we can calculate the diversity values of its language samples. The geographic location of the language sample which exhibits the largest diversity is regarded as the dispersal centre of this language family or group.

1.4 The validation for the robustness of the LVF

The estimation of the velocity field relies on the imputation strategy for missing values and three preset parameters: *k* (the number of nearest neighbours in converting the binary states into state frequencies of linguistic traits), λ (the mutation rate of the Poisson process in the parametric estimation of prestige), and *m* (the reconstruction time for calculating the velocity field). To validate the robustness of the LVF against the imputation strategy selection and the parametric setting, we perform hundreds of different statistical tests using the cosine similarity [106] and Procrustes Analysis [107,108] in both simulated and empirical validations (Supplementary Figs. 2, 3, 7, 8, 9, and 10 and Supplementary Table 3). Here, we illustrate the detailed calculation formulas and procedures in empirical validation as an example. These formulas and procedures are identical to the ones used in the simulated validation.

1.4.1 Validating the efficiency of the mode-value imputation

We calculate two velocity fields from the linguistic data with and without mode-value imputation and compare their similarity using the cosine similarity. We denote $\mathbf{V}_{\text{Im}} = \begin{bmatrix} {}^{\text{Im}}\mathbf{V}_1^T \\ {}^{\text{Im}}\mathbf{V}_2^T \\ \vdots \\ {}^{\text{Im}}\mathbf{V}_n^T \end{bmatrix}$ and $\mathbf{V}_{\text{Na}} = \begin{bmatrix} {}^{\text{Na}}\mathbf{V}_1^T \\ {}^{\text{Na}}\mathbf{V}_2^T \\ \vdots \\ {}^{\text{Na}}\mathbf{V}_n^T \end{bmatrix}$ to the velocity fields calculated from the linguistic data with and without mode-value imputation respectively. The similarity between these two velocity fields $\mathbf{V}_{\text{Im}}$ and $\mathbf{V}_{\text{Na}}$ is calculated using cosine similarity as $sim(\mathbf{V}_{\text{Im}}, \mathbf{V}_{\text{Na}}) = \frac{1}{n}\sum_{u=1}^{n}\cos\left({}^{\text{Im}}\mathbf{V}_u, {}^{\text{Na}}\mathbf{V}_u\right)$. The cosine similarity is calculated as Equation (S17). The higher value of $sim(\mathbf{V}_{\text{Im}}, \mathbf{V}_{\text{Na}})$ indicates more similarity between velocity fields calculated from the linguistic data with and without mode-value imputation. Using the permutation resampling approach [109], we examine the statistical significance of $sim(\mathbf{V}_{\text{Im}}, \mathbf{V}_{\text{Na}})$ by randomly shuffling the columns of $\mathbf{V}_{\text{Im}}$ and $\mathbf{V}_{\text{Na}}$ 500 times respectively (Supplementary Fig. 7). Moreover, we also examine the similarities among the velocity fields under different imputation strategies with the same procedure. We also examine the robustness of the principal components of the linguistic data against different imputation strategies using the Procrustes analysis. The rationale of the Procrustes analysis is to find an optimal transformation of two or more maps that maximizes the similarity of the transformed maps and to score the similarity between two optimally transformed maps.

For each language family and group, we calculate and examine the similarity between its velocity fields estimated from the linguistic data with and without mode-value imputation. Similarly, we also examine the similarities among the velocity fields and those among the PC values respectively estimated based on the linguistic data imputed by different imputation strategies. The results show that in any language family and group, the velocity fields calculated based on the linguistic data with and without mode-value imputation show significant similarities with each other (Supplementary Fig. 7). It indicates that the mode-value imputation does not affect the calculation of the velocity field. Moreover, the velocity fields and PC values respectively show significant similarities with each other under different imputation strategies. It indicates the different imputation strategies do not strongly affect the calculation of the velocity field and the PC value of the linguistic data (Supplementary Fig. 7 and Supplementary Table 3).

1.4.2 Validating the robustness of the LVF against the setting of parameter *k*

We calculate the different velocity fields under different values of k (k = 5, 10, 15, 20) and compare the similarity of each pair of the velocity fields. We denote $\mathbf{V}_{\boldsymbol{k_i}} = \begin{bmatrix} {}^{k_i}\mathbf{V}_{\mathbf{1}}^T \\ {}^{k_i}\mathbf{V}_{\mathbf{2}}^T \\ \vdots \\ {}^{k_i}\mathbf{V}_{\boldsymbol{n}}^T \end{bmatrix}$ to the velocity field calculated by setting the k nearest neighbours as $k = k_i$. The similarity between the two velocity fields $\mathbf{V}_{\boldsymbol{k_i}}$ and $\mathbf{V}_{\boldsymbol{k_j}}$ is calculated using cosine similarity as $sim\left(\mathbf{V}_{\boldsymbol{k_i}}, \mathbf{V}_{\boldsymbol{k_j}}\right) = \frac{1}{n}\sum_{u=1}^{n} \cos\left({}^{k_i}\mathbf{V}_{\boldsymbol{u}}, {}^{k_j}\mathbf{V}_{\boldsymbol{u}}\right)$. The cosine similarity is calculated as Equation (S17). The higher value of $sim\left(\mathbf{V}_{\boldsymbol{k_i}}, \mathbf{V}_{\boldsymbol{k_j}}\right)$ indicates the higher robustness of the velocity field with different settings of $k = k_i$ and $k = k_j$. We also calculate the similarity between the velocity fields $\mathbf{V}_{\boldsymbol{k_i}}^{\boldsymbol{PC}}$ and $\mathbf{V}_{\boldsymbol{k_j}}^{\boldsymbol{PC}}$ which are the projections of the $\mathbf{V}_{\boldsymbol{k_i}}$ and $\mathbf{V}_{\boldsymbol{k_j}}$ in the 2-dimensional PC space. To examine the statistical significance of $sim\left(\mathbf{V}_{\boldsymbol{k_i}}, \mathbf{V}_{\boldsymbol{k_j}}\right)$ and $sim\left(\mathbf{V}_{\boldsymbol{k_i}}^{\boldsymbol{PC}}, \mathbf{V}_{\boldsymbol{k_j}}^{\boldsymbol{PC}}\right)$ respectively, we perform the permutation test by randomly shuffling each column of the $\mathbf{V}_{\boldsymbol{k_i}}$, $\mathbf{V}_{\boldsymbol{k_j}}$, $\mathbf{V}_{\boldsymbol{k_i}}^{\boldsymbol{PC}}$, and $\mathbf{V}_{\boldsymbol{k_j}}^{\boldsymbol{PC}}$ 500 times, respectively.

For each language family or group, we calculate and examine the similarities between its velocity fields under different settings of parameter k. The results show that in any language family and group, the velocity fields in either high-dimensional or two-dimensional PC spaces show significant similarities with each other under different settings of parameter k (Supplementary Fig. 8). It indicates that the different settings of the parameter k do not affect the calculation of the velocity field in both high-dimensional and PC spaces.

1.4.3 Validating the robustness of the LVF against the setting of parameter λ

We calculate the different velocity fields under different values of λ (λ = 0.1, 0.5, 1, 5, 10) and compare the similarity of each pair of the velocity fields. We denote $\mathrm{V}_{\lambda_i} = \begin{bmatrix} {}^{\lambda_i}\mathbf{V}_1^T \\ {}^{\lambda_i}\mathbf{V}_2^T \\ \vdots \\ {}^{\lambda_i}\mathbf{V}_n^T \end{bmatrix}$ to the velocity field calculated by setting the $\lambda = \lambda_i$. The similarity between the two velocity fields $\mathbf{V}_{\lambda_i}$ and $\mathbf{V}_{\lambda_j}$ is calculated using cosine similarity as $sim\left(\mathbf{V}_{\lambda_i}, \mathbf{V}_{\lambda_j}\right) = \frac{1}{n}\sum_{u=1}^{n} \cos\left({}^{\lambda_i}\boldsymbol{V}_{\boldsymbol{u}}, {}^{\lambda_j}\boldsymbol{V}_{\boldsymbol{u}}\right)$. The cosine similarity is calculated as Equation (S17). And, the significance of $sim\left(\mathbf{V}_{\lambda_i}, \mathbf{V}_{\lambda_j}\right)$ is also examined by the permutation test with 500 times random shuffling. In addition, we calculate and examine the similarity $sim\left(\mathbf{V}_{\lambda_i}^{PC}, \mathbf{V}_{\lambda_j}^{PC}\right)$ between each pair of the velocity fields projected into the 2-dimensional space with the same procedure.

For each language family or group, we calculate and examine the similarities between its velocity fields under different settings of parameter λ. The results show that in any language family and group, the velocity fields in either high-dimensional or two-dimensional PC spaces show significant similarities with each other under different settings of parameter λ (Supplementary Fig. 9). It indicates that the different settings of the parameter λ do not affect the calculation of the velocity field in both high-dimensional and PC spaces.

1.4.4 Validating the robustness of the LVF against the setting of parameter *m*

We calculate the different language velocity fields under different values of m (m = 1, 3, 5, 7, and 9), and compare the similarity of each pair of the velocity fields. We denote $\mathbf{V}_{m_i} = \begin{bmatrix} {}^{m_i}\mathbf{V}_1^T \\ {}^{m_i}\mathbf{V}_2^T \\ \vdots \\ {}^{m_i}\mathbf{V}_n^T \end{bmatrix}$ to the velocity field calculated by setting the reconstruction time as $m = m_i$. The similarity between the two velocity fields $\mathbf{V}_{m_i}$ and $\mathbf{V}_{m_j}$ is calculated using cosine similarity as $sim\left(\mathbf{V}_{m_i}, \mathbf{V}_{m_j}\right) = \frac{1}{n}\sum_{u=1}^{n} \cos\left({}^{m_i}\mathbf{V}_{\boldsymbol{u}}, {}^{m_j}\mathbf{V}_{\boldsymbol{u}}\right)$. The cosine similarity is calculated as Equation (S17). We also perform the permutation test to examine the statistical significance of the similarity $sim\left(\mathbf{V}_{m_i}, \mathbf{V}_{m_j}\right)$ by randomly shuffling the columns of the $\mathbf{V}_{m_i}$ and $\mathbf{V}_{m_j}$ 500 times, respectively. For the projection of the $\mathbf{V}_{m_i}$ and $\mathbf{V}_{m_j}$ in the two-dimensional PC space ($\mathbf{V}_{m_i}^{PC}$ and $\mathbf{V}_{m_j}^{PC}$), we also calculate and examine their similarity $sim\left(\mathbf{V}_{m_i}^{PC}, \mathbf{V}_{m_j}^{PC}\right)$ with the same procedures.

For each language family or group, we calculate and examine the similarities between its velocity fields

under different settings of reconstrued time. The results show that in any language family and group, the velocity fields in either high-dimensional or two-dimensional PC spaces show significant similarities with each other under different settings of reconstruction time (Supplementary Fig. 10). It indicates that the different settings of reconstruction time do not affect the calculation of the velocity field in both high-dimensional and PC spaces.

Supplementary References

1 Koile, E., Chechuro, I., Moroz, G. & Daniel, M. Geography and language divergence: The case of Andic languages. *Plos one* **17**, e0265460 (2022).

2 Bouckaert, R. *et al.* Mapping the origins and expansion of the Indo-European language family. *Science* **337**, 957-960 (2012).

3 Grollemund, R. *et al.* Bantu expansion shows that habitat alters the route and pace of human dispersals. *Proceedings of the National Academy of Sciences* **112**, 13296-13301 (2015).

4 Bouckaert, R. R., Bowern, C. & Atkinson, Q. D. The origin and expansion of Pama–Nyungan languages across Australia. *Nature Ecology & Evolution* **2**, 741-749 (2018).

5 Walker, R. S. & Ribeiro, L. A. Bayesian phylogeography of the Arawak expansion in lowland South America. *Proceedings of the Royal Society B: Biological Sciences* **278**, 2562-2567 (2011).

6 Felsenstein, J. Phylogenies and the comparative method. *The American Naturalist* **125**, 1-15 (1985).

7 Freckleton, R. P., Harvey, P. H. & Pagel, M. Phylogenetic analysis and comparative data: a test and review of evidence. *The American Naturalist* **160**, 712-726 (2002).

8 Currie, T. E., Meade, A., Guillon, M. & Mace, R. Cultural phylogeography of the Bantu Languages of sub-Saharan Africa. *Proceedings of the Royal Society B: Biological Sciences* **280**, 20130695 (2013).

9 La Manno, G. *et al.* RNA velocity of single cells. *Nature* **560**, 494-498 (2018).

10 Zhang, M. & Gong, T. Principles of parametric estimation in modeling language competition. *Proceedings of the National Academy of Sciences* **110**, 9698-9703 (2013).

11 Gray, R. D. & Atkinson, Q. D. Language-tree divergence times support the Anatolian theory of Indo-European origin. *Nature* **426**, 435-439 (2003).

12 Zhang, M., Yan, S., Pan, W. & Jin, L. Phylogenetic evidence for Sino-Tibetan origin in northern China in the Late Neolithic. *Nature* **569**, 112-115 (2019).

13 Choudhuri, S. *Bioinformatics for beginners: genes, genomes, molecular evolution, databases and analytical tools*. (Elsevier, 2014).

14 Lewis, P. O. A genetic algorithm for maximum-likelihood phylogeny inference using nucleotide sequence data. *Molecular biology and evolution* **15**, 277-283 (1998).

15 Felsenstein, J. Evolutionary trees from DNA sequences: a maximum likelihood approach. *Journal of molecular evolution* **17**, 368-376 (1981).

16 Goldman, N. Statistical tests of models of DNA substitution. *Journal of molecular evolution* **36**, 182-198 (1993).

17 Gray, R. D., Drummond, A. J. & Greenhill, S. J. Language phylogenies reveal expansion pulses and pauses in Pacific settlement. *Science* **323**, 479-483 (2009).

18 Neureiter, N., Ranacher, P., van Gijn, R., Bickel, B. & Weibel, R. Can Bayesian phylogeography reconstruct migrations and expansions in linguistic evolution? *Royal Society open science* **8**, 201079 (2021).

19 Efron, B. *The jackknife, the bootstrap and other resampling plans*. (SIAM, 1982).

20 Wichmann, S. & Rama, T. Testing methods of linguistic homeland detection using synthetic data. *Philosophical Transactions of the Royal Society B* **376**, 20200202 (2021).

21 Carter, C. Great circle distances. *SiRF White Paper* (2002).

22 Jobling, M. A., Hurles, M. & Tyler-Smith, C. *Human evolutionary genetics: origins, peoples and disease*. (Garland Science, 2019).

23 Bocquet-Appel, J.-P. When the world's population took off: the springboard of the Neolithic Demographic Transition. *Science* **333**, 560-561 (2011).

24 Diamond, J. & Bellwood, P. Farmers and their languages: the first expansions. *Science* **300**, 597-603 (2003).

25 Janhunen, J. Manchuria: An Ethnic History. *Finno-Ugrian Society* (1996).

26 Holden, C. J. Bantu language trees reflect the spread of farming across sub-Saharan Africa: a maximum-parsimony analysis. *Proceedings of the Royal Society of London. Series B: Biological Sciences* **269**, 793-799 (2002).

27 Chang, W., Hall, D., Cathcart, C. & Garrett, A. Ancestry-constrained phylogenetic analysis supports the Indo-European steppe hypothesis. *Language*, 194-244 (2015).

28 Mallory, J. P. & Adams, D. Q. *The Oxford introduction to proto-Indo-European and the proto-Indo-European world*. (Oxford University Press on Demand, 2006).

29 Anthony, D. W. *et al.* The" Kurgan Culture," Indo-European origins, and the domestication of the horse: a reconsideration [and comments and replies]. *Current Anthropology* **27**, 291-313 (1986).

30 Cardona, G., Hoenigswald, H. M. & Senn, A. *Indo-European and Indo-Europeans*. (University of Pennsylvania Press Philadelphia, 1970).

31 Gimbutas, M. The first wave of Eurasian pastoralists into Copper Age Europe. *Journal of Indo-*

European Studies Washington, DC **5**, 277-338 (1977).

32 Levine, M., Rassamakin, Y., Kislenko, A. & Tatarintseva, N. *Late prehistoric exploitation of the Eurasian steppe*. (McDonald Inst of Archeological, 1999).

33 Renfrew, C. Archaeology and Language (Jonathan Cape, London). (1987).

34 Dolgopolsky, A. The Indo-European homeland and lexical contacts of Proto-Indo-European with other languages. *Mediterranean Language Review* **3**, 7-31 (1987).

35 Krantz, G. S. *Geographical development of European languages*. Vol. 26 (Peter Lang Pub Incorporated, 1988).

36 Renfrew, C. Time depth, convergence theory, and innovation in Proto-Indo-European:'Old Europe'as a PIE linguistic area. *Journal of Indo-European Studies* **27**, 257 (1999).

37 Watkins, C. *The American heritage dictionary of Indo-European roots*. (Houghton Mifflin Harcourt, 2000).

38 Mallory, J. P. J. A., Theoretical, L. I. & orientations, m. The homelands of the Indo-Europeans. *Archaeology and Language I: Theoretical methodological orientations*, 93-121 (1997).

39 Anthony, D. W. Horse, wagon & chariot: Indo-European languages and archaeology. *Antiquity* **69**, 554-565 (1995).

40 Matisoff, J. A. Sino-Tibetan linguistics: present state and future prospects. *Annual review of anthropology* **20**, 469-504 (1991).

41 Handel, Z. What is Sino-Tibetan? Snapshot of a Field and a Language Family in Flux. *Language and Linguistics Compass* **2**, 422-441 (2008).

42 Benedict, B. P. K., Benedict, P. K. & Matisoff, J. A. *Sino-Tibetan: a conspectus*. (CUP Archive, 1972).

43 Matisoff, J. A. *Handbook of Proto-Tibeto-Burman: system and philosophy of Sino-Tibetan reconstruction*. (Univ of California Press, 2003).

44 Aĭkhenval'd, A. I. U. r. e. & Ajchenval'd, A. J. *Areal diffusion and genetic inheritance: Problems in comparative linguistics*. (Oxford University Press on Demand, 2001).

45 Blench, R. & Post, M. W. Rethinking Sino-Tibetan phylogeny from the perspective of North East Indian languages. *Trans-Himalayan Linguistics* **266**, 71-104 (2014).

46 Owen-Smith, T. & Hill, N. *Trans-Himalayan linguistics: historical and descriptive linguistics of the Himalayan area*. Vol. 266 (Walter de Gruyter, 2013).

47 Peiros, I. & Starostin, S. A. *A Comparative Vocabulary of Five Sino-Tibetan Languages: Velars, uvulars and laringals*. (University of Melbourne, Department of Linguistics and Applied Linguistics, 1996).

48 Peiros, I. *Comparative Linguistics in Southeast Asia*. (Pacific Linguistics, Research School of Pacific and Asian Studies, The …, 1998).

49 Shafer, R. Classification of the Sino-Tibetan languages. *Word* **11**, 94-111 (1955).

50 Thurgood, G. & LaPolla, R. J. *The sino-tibetan languages*. (Taylor & Francis, 2016).

51 Sprague, D. The Peopling of East Asia: Putting Together Archaeology, Linguistics and Genetics. *ANTHROPOLOGICAL SCIENCE* **115**, 73-78 (2007).

52 Van Driem, G. Implications for population geneticists, archaeologists and prehistorians. *The peopling of East Asia: putting together archaeology, linguistics and genetics*, 81 (2005).

53 Fei, X. On the problem of distinguishing nationalities in China. *Soc. Sci. China* **1**, 158-174 (1980).

54 Adler, P. J. & Pouwels, R. L. *World civilizations: volume i: to 1700*. (Cengage Learning, 2014).

55 Falola, T. & Usman, A. Movements, Borders, and Identities in Africa. *African Diaspora Archaeology Newsletter* **12**, 37 (2009).

56 Russell, T., Silva, F. & Steele, J. Modelling the spread of farming in the Bantu-speaking regions of Africa: an archaeology-based phylogeography. *PLoS One* **9**, e87854 (2014).

57 Neumann, K. *et al.* First farmers in the Central African rainforest: A view from southern Cameroon. *Quaternary International* **249**, 53-62 (2012).

58 Vincens, A. *et al.* Changement majeur de la végétation du lac Sinnda (vallée du Niari, Sud Congo) consécutif à l'assèchement climatique holocène supérieur: apport de la palynologie. *Comptes Rendus à l'Academie des Sciences de Paris* **318**, 1521-1526 (1994).

59 Bostoen, K. *et al.* Middle to late Holocene Paleoclimatic change and the early Bantu expansion in the rain forests of Western Central Africa. *Current Anthropology* **56**, 354-384 (2015).

60 Vincens, A., Schwartz, D., Bertaux, J., Elenga, H. & de Namur, C. Late Holocene climatic changes in western equatorial Africa inferred from pollen from Lake Sinnda, southern Congo. *Quaternary Research* **50**, 34-45 (1998).

61 Maley, J. La destruction catastrophique des forêts d'Afrique centrale survenue il ya environ 2500 ans exerce encore une influence majeure sur la répartition actuelle des formations végétales. *Systematics and Geography of Plants*, 777-796 (2001).

62 Maley, J. & Willis, K. Did a savanna corridor open up across the Central African forests 2500 years ago. *CoForChange* (2010).

63 White, F. The Guineo-Congolian Region and its relationships to other phytochoria. *Bulletin du Jardin botanique national de Belgique/Bulletin van de Nationale Plantentuin van Belgie*, 11-55 (1979).

64 Gond, V. *et al.* Vegetation structure and greenness in Central Africa from Modis multi-temporal data. *Philosophical Transactions of the Royal Society B: Biological Sciences* **368**, 20120309 (2013).

65 Doumenge, C., Gonmadje, C., Doucet, J.-L. & Maley, J. in *20th AETFAT Congress.*

66 Aikhenvald, A. Y. in *Encyclopedia of Languages and Linguistics* (ed Keith Brown) (Elsevier, 2006).

67 Olsen, K. M. & Schaal, B. A. Microsatellite variation in cassava (Manihot esculenta, Euphorbiaceae) and its wild relatives: further evidence for a southern Amazonian origin of domestication. *American journal of botany* **88**, 131-142 (2001).

68 Pearsall, D. M. in *The handbook of South American archaeology* 105-120 (Springer, 2008).

69 Baker, M. Foundations of the Age-Area Hypothesis. *Humanities and Social Sciences Communications* **8**, 1-17 (2021).

70 Wichmann, S., Müller, A. & Velupillai, V. Homelands of the world's language families: A quantitative approach. *Diachronica* **27**, 247-276 (2010).

71 Lemey, P., Rambaut, A., Drummond, A. J. & Suchard, M. A. Bayesian phylogeography finds its roots. *PLoS computational biology* **5**, e1000520 (2009).

72 Sapir, E. *Time perspective in aboriginal American culture: a study in method*. Vol. 90 (Government Printing Bureau, 1916).

73 Kitchen, A., Ehret, C., Assefa, S. & Mulligan, C. Bayesian phylogenetic analysis of Semitic languages identifies an Early Bronze Age origin of Semitic in the Near East. *Proceedings of the Royal Society B: Biological Sciences* **276**, 2703-2710 (2009).

74 Nettle, D. Explaining global patterns of language diversity. *Journal of anthropological archaeology* **17**, 354-374 (1998).

75 Greenhill, S. in *The Routledge handbook of historical linguistics* (Routledge Taylor & Francis Group, 2015).

76 Epps, P. Forthcoming. Amazonian linguistic diversity and its sociocultural correlates. *Language dispersal, diversification, and contact: a global perspective*.

77 Derungs, C., Köhl, M., Weibel, R. & Bickel, B. Environmental factors drive language density more in food-producing than in hunter–gatherer populations. *Proceedings of the Royal Society B* **285**, 20172851 (2018).

78 Pacheco Coelho, M. T. *et al.* Drivers of geographical patterns of North American language diversity. *Proceedings of the Royal Society B* **286**, 20190242 (2019).

79 François, A. in *Trees, waves and linkages: Models of language diversification* (Routledge, 2014).

80 Greenhill, S. J., Currie, T. E. & Gray, R. D. Does horizontal transmission invalidate cultural phylogenies? *Proceedings of the Royal Society B: Biological Sciences* **276**, 2299-2306 (2009).

81 Lynch, J., Ross, M. & Crowley, T. *The oceanic languages*. Vol. 1 (Psychology Press, 2002).

82 Masica, C. P. *The indo-aryan languages*. (Cambridge University Press, 1993).

83 Al-Jallad, A. On the genetic background of the Rbbl bn Hfˁm grave inscription at Qaryat al-Fāw1. *Bulletin of the School of Oriental and African Studies* **77**, 445-465 (2014).

84 Grenoble, L. A. *Language policy in the Soviet Union*. Vol. 3 (Springer Science & Business Media, 2003).

85 Norman, J. *Chinese*. (Cambridge University Press, 1988).

86 Alkire, T. & Rosen, C. *Romance languages: A historical introduction*. (Cambridge University Press, 2010).

87 Harbert, W. *The Germanic Languages*. (Cambridge University Press, 2006).

88 Sussex, R. & Cubberley, P. *The slavic languages*. (Cambridge University Press, 2006).

89 Galbis, A. & Maestre, M. *Vector analysis versus vector calculus*. (Springer Science & Business Media, 2012).

90 Sohn, B.-J., Yeh, S.-W., Lee, A. & Lau, W. K. Regulation of atmospheric circulation controlling the tropical Pacific precipitation change in response to CO2 increases. *Nature communications* **10**, 1-8 (2019).

91 Mazzoli, M. *et al.* Field theory for recurrent mobility. *Nature communications* **10**, 1-10 (2019).

92 Fort, J. Demic and cultural diffusion propagated the Neolithic transition across different regions of Europe. *Journal of the Royal Society interface* **12**, 20150166 (2015).

93 Yang, Z. Maximum-likelihood estimation of phylogeny from DNA sequences when substitution rates differ over sites. *Molecular biology and evolution* **10**, 1396-1401 (1993).

94 Penny, D., McComish, B. J., Charleston, M. A. & Hendy, M. D. Mathematical elegance with biochemical realism: the covarion model of molecular evolution. *Journal of Molecular Evolution* **53**, 711-723 (2001).

95 *The ASJP Database (version 19)*. (2020).

96 Borg, I. & Groenen, P. J. *Modern multidimensional scaling: Theory and applications*. (Springer Science & Business Media, 2005).

97 Schölkopf, B., Smola, A. & Müller, K.-R. Nonlinear component analysis as a kernel eigenvalue problem. *Neural computation* **10**, 1299-1319 (1998).

98 Abrams, D. M. & Strogatz, S. H. Modelling the dynamics of language death. *Nature* **424**, 900-900 (2003).

99 Cho, A. Constructing phylogenetic trees using maximum likelihood. (2012).

100 Butcher, J. C. A history of Runge-Kutta methods. *Applied numerical mathematics* **20**, 247-260 (1996).

101 Soetaert, K., Petzoldt, T. & Setzer, R. W. Solving differential equations in R: package deSolve. *Journal of statistical software* **33**, 1-25 (2010).

102 Ouda, R. & Hart, P. Pattern classification and scene analysis wiley. *New York* (1973).

103 Hennig, C. & Imports, M. Package 'fpc'. *Flexible Procedures for Clustering* (2015).

104 Blust, R. The Austronesian homeland: a linguistic perspective. *Asian Perspectives* **26**, 45-67 (1984).

105 Shannon, C. E. A mathematical theory of communication. *The Bell system technical journal* **27**, 379-423 (1948).

106 Singhal, A. Modern information retrieval: A brief overview. *IEEE Data Eng. Bull.* **24**, 35-43 (2001).

107 Kent, J., Bibby, J. & Mardia, K. *Multivariate analysis*. (Academic Press Amsterdam, 1979).

108 Peres-Neto, P. R. & Jackson, D. A. How well do multivariate data sets match? The advantages of a Procrustean superimposition approach over the Mantel test. *Oecologia* **129**, 169-178 (2001).

109 Moore, J. H. Bootstrapping, permutation testing and the method of surrogate data. *Physics in Medicine Biology* **44**, L11 (1999).

Supplementary Tables

Supplementary Table 1. Description of datasets of the four language cases

Language case	Number of samples	Missing values	Estimated divergence time (years BP)	Phylogeographic reconstruction (Yes/No)	Reference
**Indo-European**	103	Yes	~7,000 – 10,000	YES	[2]
**Sino-Tibetan**	109	Yes	~4,000 – 7,800	YES	[12]
**Bantu**	420	Yes	~4,700 – 5,000	YES	[3]
**Arawak**	60	Yes	< 5,000	YES	[5]

Supplementary Table 2. The inferred coordinates of the dispersal centres of four language cases

Language case	Longitude±SD	Latitude±SD
**Indo-European**	40.14±8.41	39.92±6.01
**Sino-Tibetan**	105.60±1.09	34.85±5.63
**Bantu**	13.15±0.16	3.00±0.28
**Arawak**	-66.00±1.84	-11.50±3.26

* The Standard Deviations (SDs) of longitude and latitude are calculated based on the Jackknife resampling.

Supplementary Table 3. The similarities between the principal components of linguistic data under different imputation approaches

Language case	mode vs zero		mode vs frequency		frequency vs zero	
	cor	*p*-value	*cor*	*p*-value	*cor*	*p*-value
**Indo-European**	0.9989	0.001	0.9997	0.001	0.9976	0.001
**Sin-Tibetan**	0.9996	0.001	0.9994	0.001	0.9991	0.001
**Bantu**	0.9540	0.001	0.9986	0.001	0.9463	0.001
**Arawak**	0.9964	0.001	0.9989	0.001	0.9943	0.001

* The *cor* denotes the correlation ranging from 0 to 1, which is calculated using the Procrustes Analysis. A value closer to 1 denotes that the principal components calculated under different imputation approaches are more similar. The *p*-value is calculated based on the one-sided permutation test with 1000 times random shuffle.

Supplementary Figures

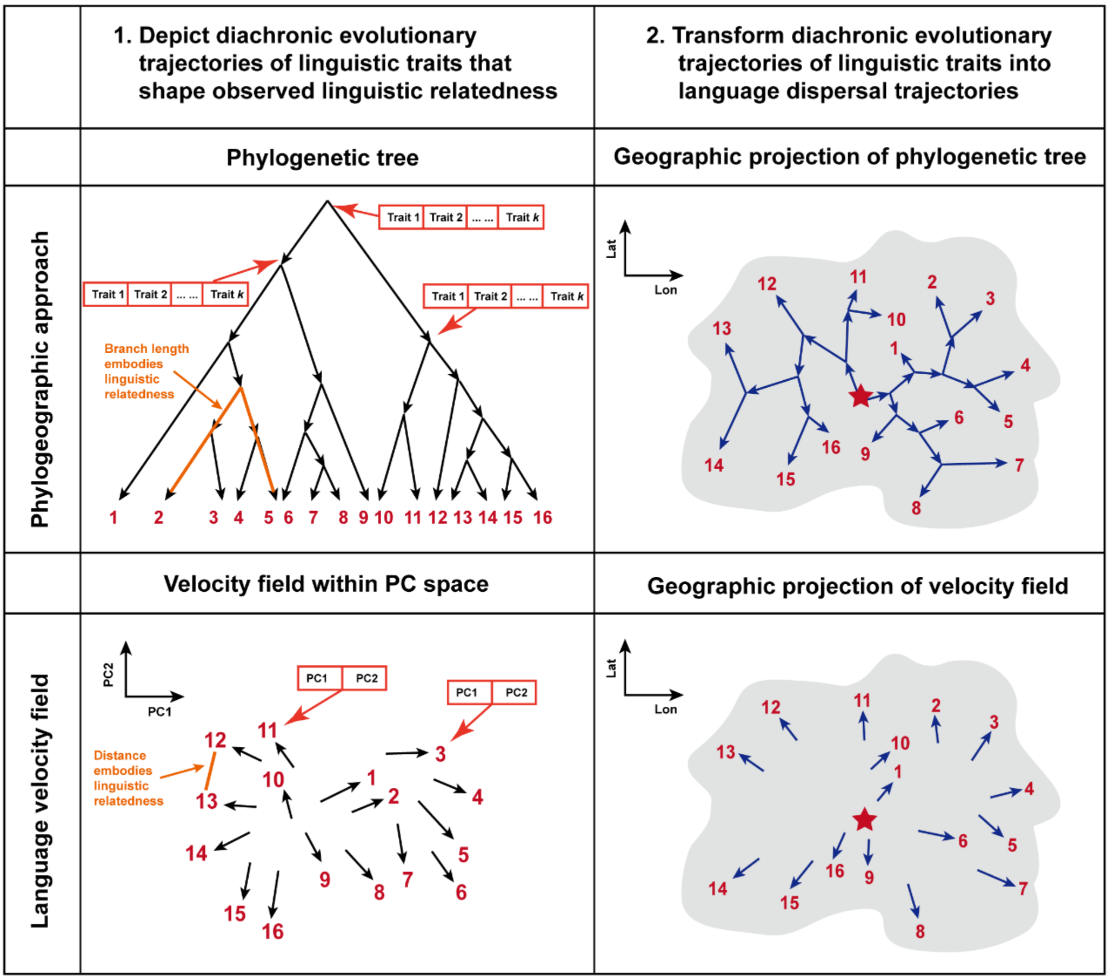

Supplementary Fig. 1: The consistency and inconsistency between LVF and phylogeographic approach. Both LVF and phylogeographic approach entail two major steps to infer language dispersal patterns. The first is to depict the diachronic evolutionary trajectories of linguistic traits that shape the observed linguistic relatedness among language samples. The second is to transform these diachronic evolutionary trajectories of linguistic traits into language dispersal trajectories. In the phylogenetic tree, each language sample is determined by *k* linguistic traits. In the velocity field within PC space, each language sample is determined by PC1 and PC2 which are rearranged from the *k* linguistic traits through the PCA algorithm. The red number denotes each language sample. The black arrow signifies the evolutionary direction of linguistic traits in each language sample. The blue arrow represents the dispersal direction of each language sample. The red star denotes the estimated dispersal centre.

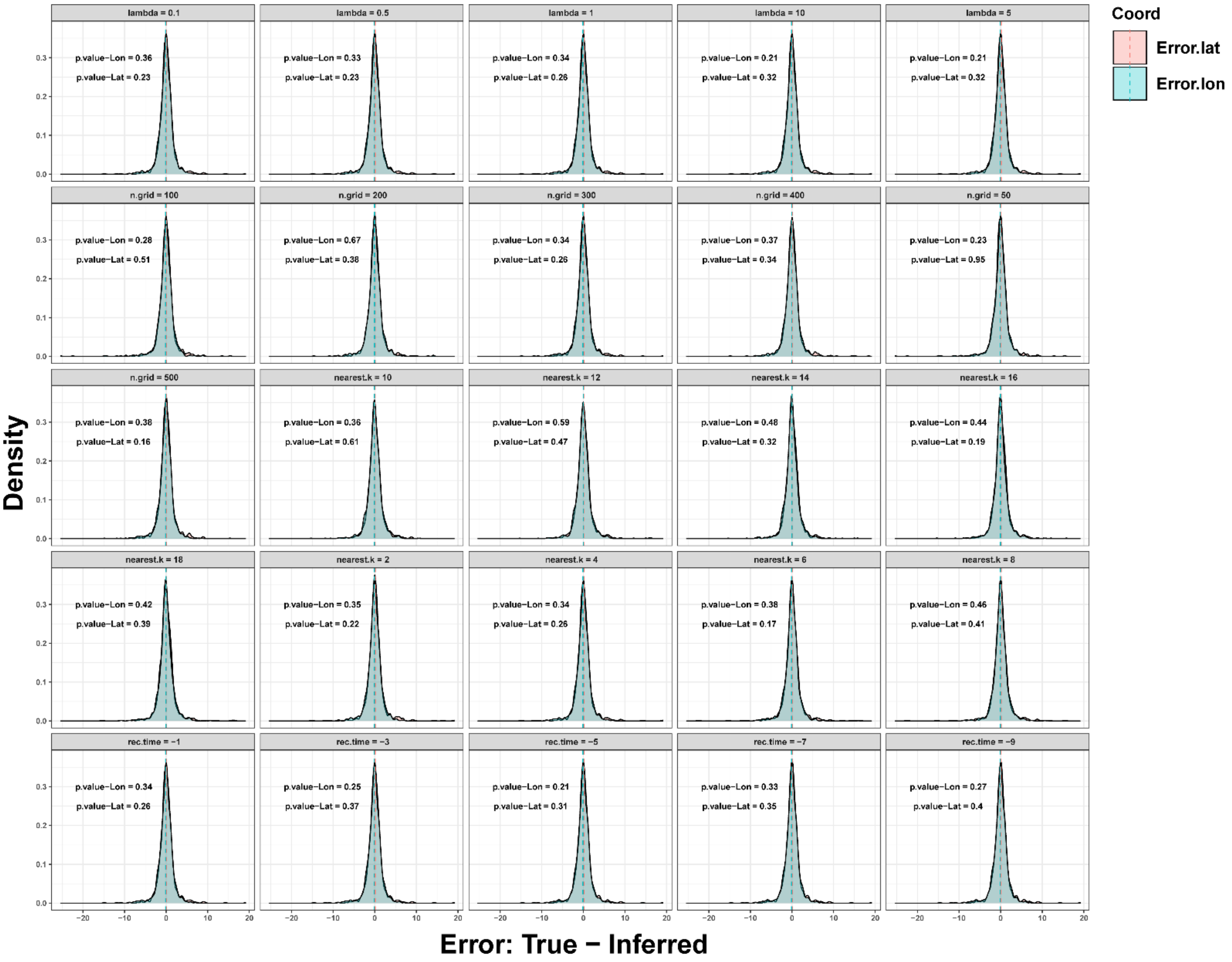

Supplementary Fig. 2: The simulated validations for the effectiveness of the LVF under different parametric settings. The probability density plot demonstrates the distributions of the errors in longitude and latitude respectively between the true/given and inferred language dispersal centres estimated from 1,000 simulated datasets under different parametric settings. These parameters are the number of the grid points *n.grid* (*n.grid* = 50, 100, 200, 300, 400, and 500); the number of the nearest neighbours k (k = 2, 4, 6, …, and 18); mutation rate of Poisson process λ (λ = 0.1, 0.5, 1, 5, and 10); reconstruction time m (m = 1, 3, 5, 7, and 9). We set the default parametric values as *n.grid* = 300, k = 4, λ = 1, and m = 1 when varying across the settings of these parameters respectively. The black texts are the p-values of the statistical significance of the errors in terms of longitude and latitude derived from the two-sided Wilcoxon rank-sum test. p-value > 0.05 denotes the statistical non-significance of the error (significantly equal to 0). The Source Data and Codes for generating this figure are available.

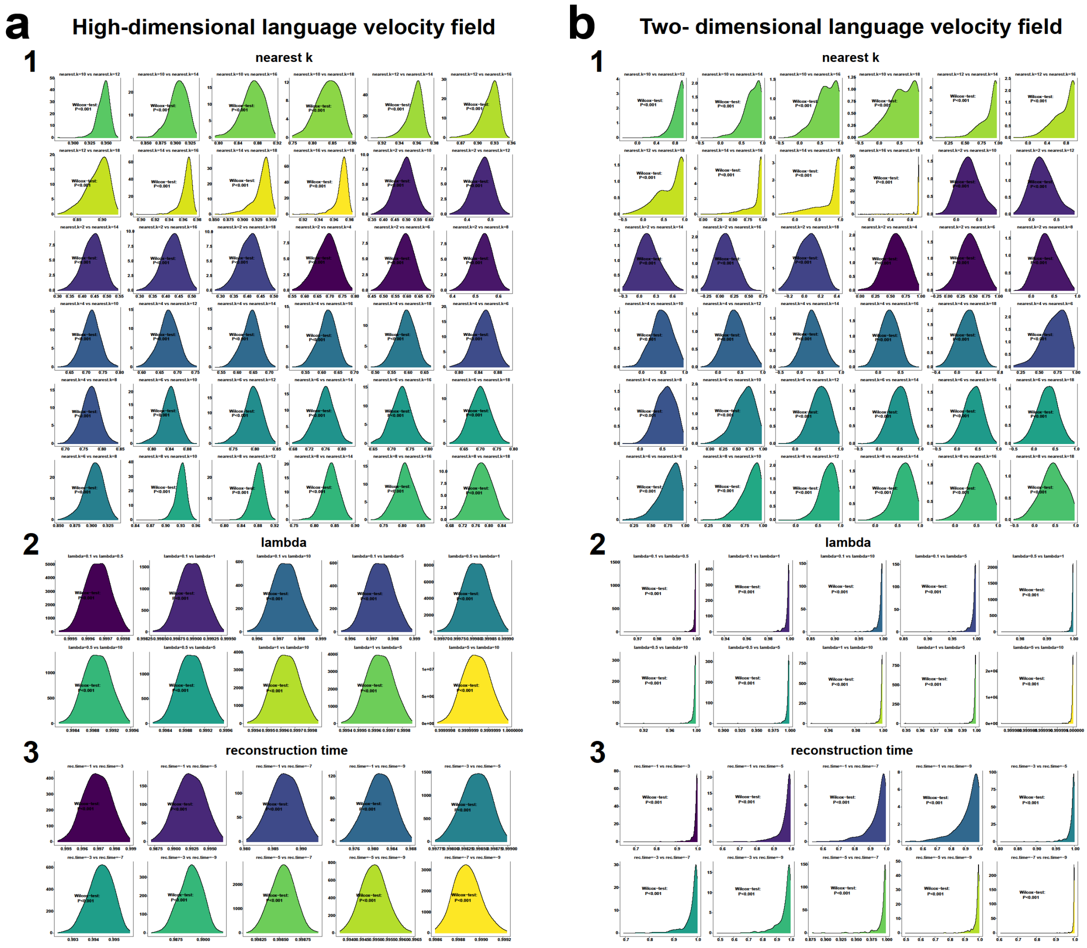

Supplementary Fig. 3: The simulated validations for the robustness of the LVF under different parametric settings. Each panel displays a probability density plot that demonstrates the distribution of the average cosine similarity between two velocity fields within high-dimensional or two-dimensional spaces estimated from 1,000 simulated datasets under a pair of values for a specific parameter. The black text is the *p*-value of the statistical significance of this average similarity derived from the two-sided Wilcoxon rank-sum test. *p*-value < 0.05 denotes the statistical significance of this average similarity (significantly not equal to 0). (a1) Each panel demonstrates the distribution within high-dimensional space estimated under one of the pairwise combinations of nearest neighbours k (k = 2, 4, 6, …, and 18). (a2) Each panel demonstrates the distribution within high-dimensional space estimated under one of the pairwise combinations of mutation rate λ (λ = 0.1, 0.5, 1, 5, and 10). (a3) Each panel demonstrates the distribution within high-dimensional space estimated under one of the pairwise combinations of reconstruction time m (m = 1, 3, 5, 7, and 9). (b1) Each panel demonstrates the distribution within two-dimensional space estimated under one of the pairwise combinations of nearest neighbours k (k = 2, 4, 6, …, and 18). (b2) Each panel demonstrates the distribution within two-dimensional space estimated under one of the pairwise combinations of mutation rate λ (λ = 0.1, 0.5, 1, 5, and 10). (b3) Each panel demonstrates the distribution within two-dimensional space estimated under one of the pairwise combinations of reconstruction time m (m = 1, 3, 5, 7, and 9). We set the default parametric values as $k = 4$, $\lambda = 1$, and $m = 1$ when varying across the settings of these parameters respectively. The Source Data and Codes for generating this figure are available.

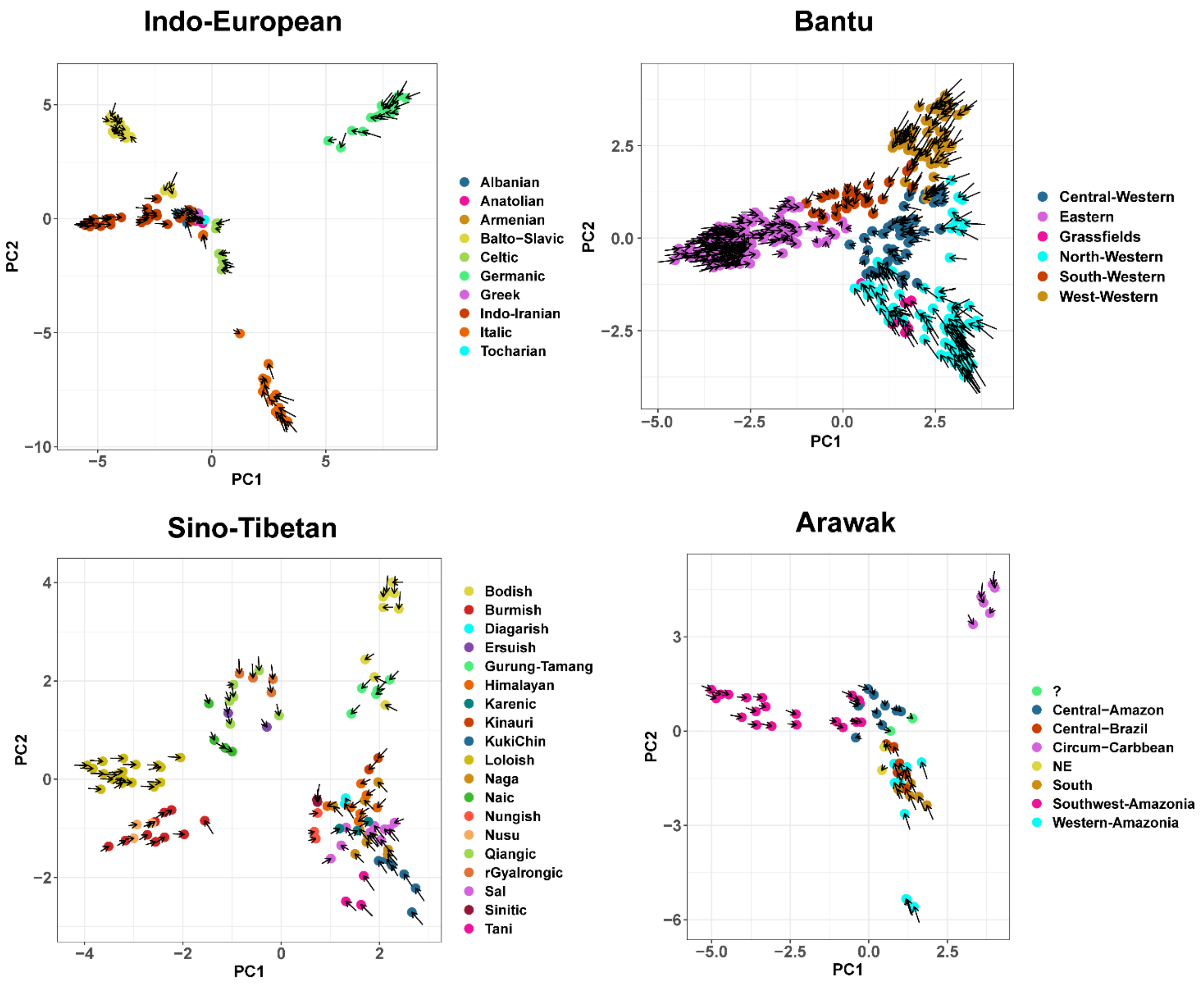

Supplementary Fig. 4: The velocity fields of four language families and groups in the PC space. The velocity fields of four language families and groups are projected using the PCA algorithm from the high-dimensional space to the two-dimensional PC space. The black arrow denotes the velocity vector of each language sample. The Source Data and Codes for generating this figure are available.

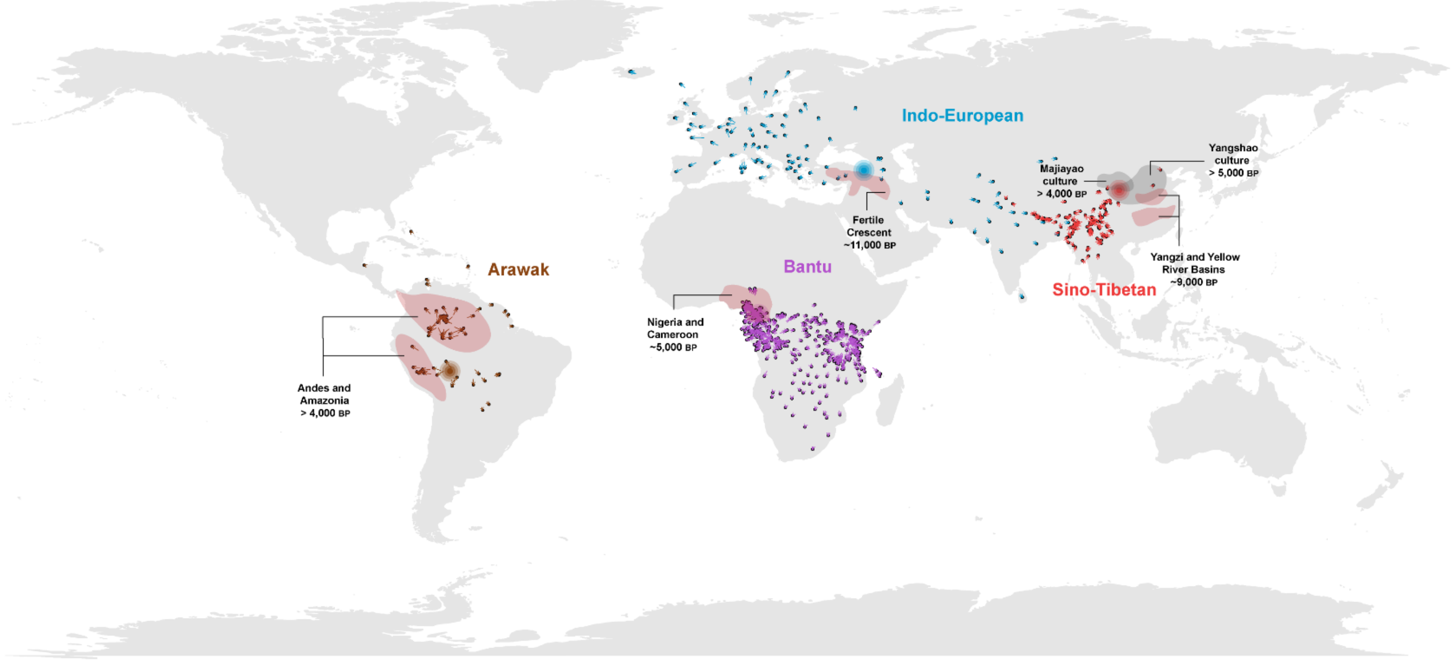

Supplementary Fig. 5: The velocity fields of four language families and groups within geographic space. The velocity fields of the four language families and groups projected from the PC space into the geographic space using the kernel projection. The arrow signifies the velocity vector of each language sample. The length of each arrow has been adjusted using the spatial smoothing approach. The direction of the arrow represents the change in the geographic positions of each language sample from the past to the present. And, the larger length of the arrow denotes the more rapid change while the lower length denotes the slower. The pale red polygon denotes the ancient agricultural homeland, whereas the black text signifies its corresponding name and origin time. The grey polygon represents the geographic range of the known Neolithic culture, whereas the black text signifies its corresponding name and origin time. The grey base world map is generated using the map function of the ape package in R (4.3.1). The Source Data and Codes for generating this figure are available.

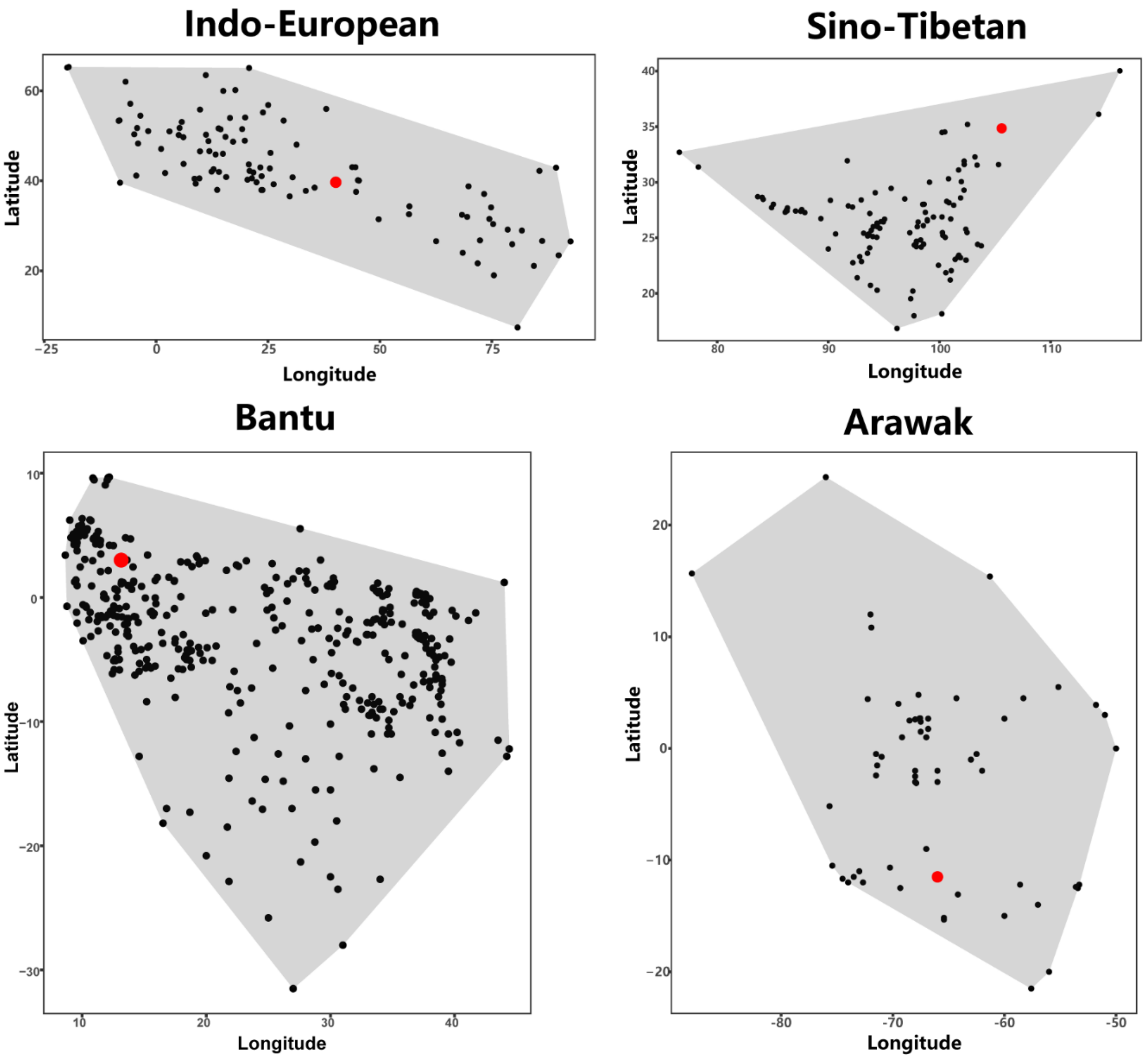

Supplementary Fig. 6: The convex hull of the language samples in four language families and groups. The black point denotes each language sample. The red point represents the inferred dispersal centre of each language family or group. The gray polygon denotes the convex hull generated from the geographic coordinates of language samples within each language family or group. The Source Data and Codes for generating this figure are available.

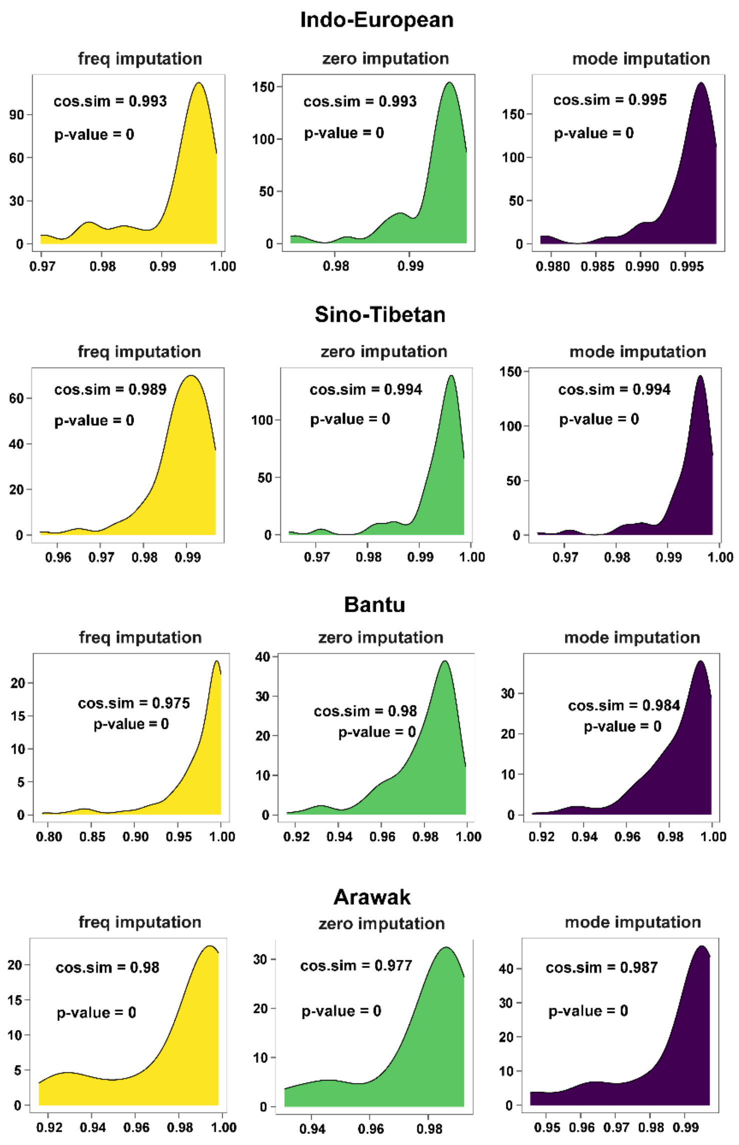

Supplementary Fig. 7: The empirical validations for the robustness of the LVF against the selection of the imputation approaches. The probability density plot demonstrates the distribution of the cosine similarity between two corresponding velocity vectors in a velocity field pair estimated with and without the imputation of missing values. Three imputation approaches are frequency-value imputation, zero-value imputation, and mode-value imputation, and they impute the missing values for each trait with its state frequency, zero value, and mode value respectively. The black texts show the average similarity between the velocity vectors in two velocity fields and the *p*-value for the statistical significance of this average similarity derived from the one-sided permutation test (Permutation Times = 500). The average similarity value ranges from 0 to 1, where the value closer to 1 denotes that these two velocity fields are more similar. p-value < 0.05 denotes the statistical significance of the average similarity (significantly not equal to 0). The Source Data and Codes for generating this figure are available.

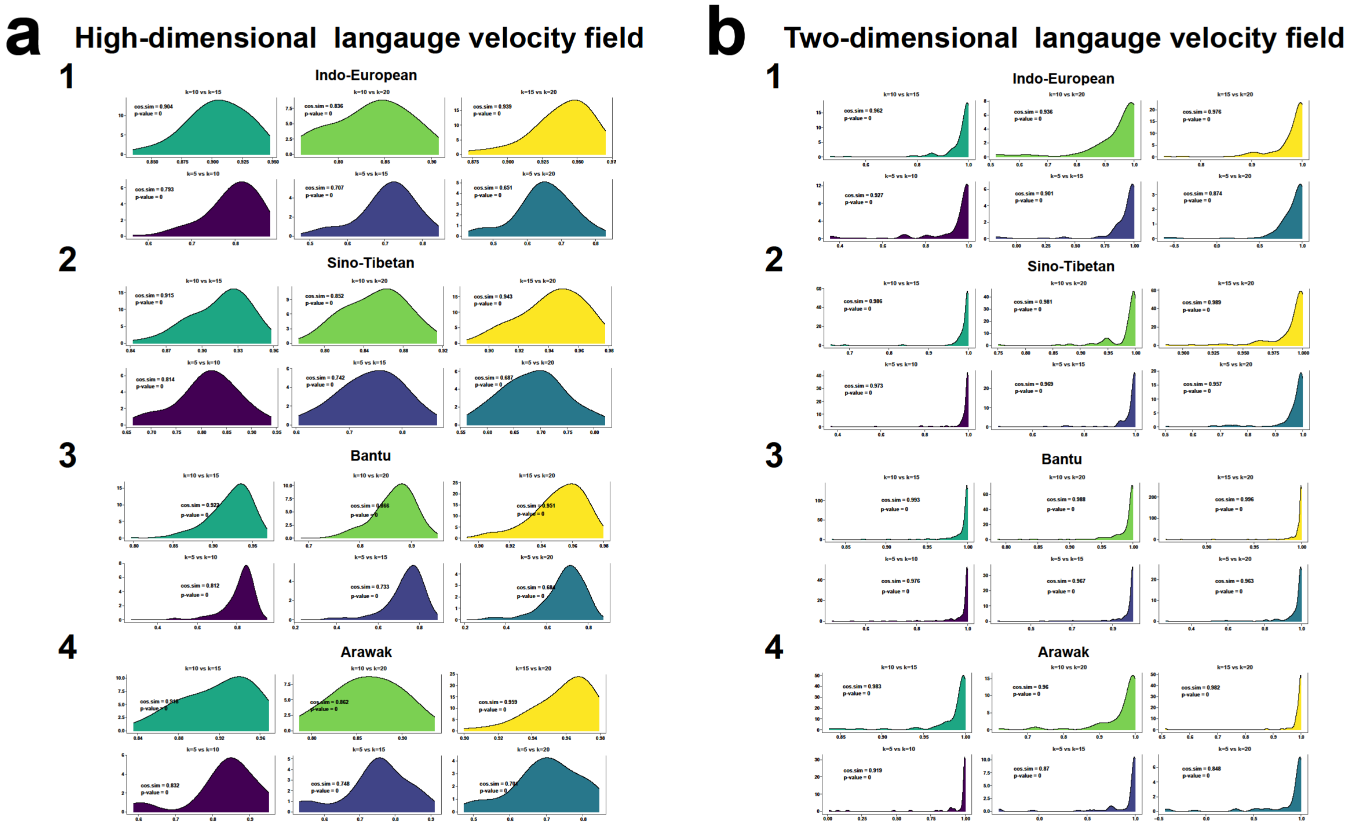

Supplementary Fig. 8: The empirical validations for the robustness of the LVF against the setting of the *k*-nearest neighbours. Each panel displays a probability density plot that demonstrates the distribution of the cosine similarity between two corresponding velocity vectors in two velocity fields within high-dimensional or two-dimensional spaces estimated under one of the pairwise combinations of k (k = 5, 10, 15, 20) in each language case. The black texts are the average similarity between the velocity vectors in two velocity fields and the p-value for the statistical significance of this average similarity derived from the one-sided permutation test (Permutation Times = 500). The average similarity ranges from 0 to 1, where the value closer to 1 denotes that these two velocity fields are more similar. p-value < 0.05 denotes the statistical significance of the average similarity (significantly not equal to 0). (a1) Each panel demonstrates the distribution within high-dimensional space for the Indo-European language case. (a2) Each panel demonstrates the distribution within high-dimensional space for the Sino-Tibetan language case. (a3) Each panel demonstrates the distribution within high-dimensional space for the Bantu language case. (a4) Each panel demonstrates the distribution within high-dimensional space for the Arawak language case. (b1) Each panel demonstrates the distribution within two-dimensional space for the Indo-European language case. (b2) Each panel demonstrates the distribution within two-dimensional space for the Sino-Tibetan language case. (b3) Each panel demonstrates the distribution within two-dimensional space for the Bantu language case. (b4) Each panel demonstrates the distribution within two-dimensional space for the Arawak language case. We set the default parametric values as $\lambda = 1$ and $m = 1$ when varying across the settings of k. The Source Data and Codes for generating this figure are available.

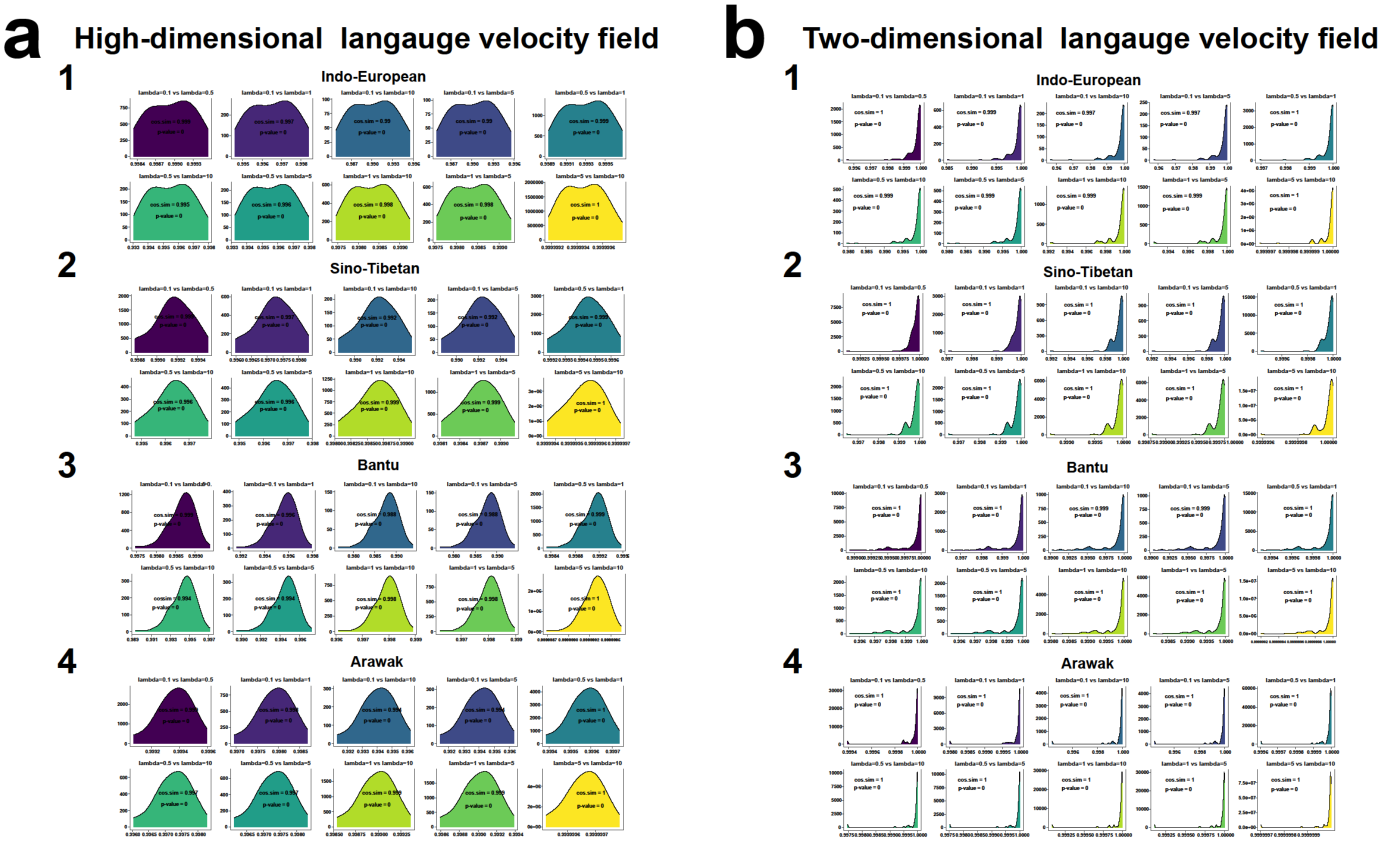

Supplementary Fig. 9: The empirical validations for the robustness of the LVF against the setting of the mutation rate in the Poisson process. Each panel displays a probability density plot that demonstrates the distribution of the cosine similarity between two corresponding velocity vectors in two velocity fields within high-dimensional or two-dimensional spaces estimated under one of the pairwise combinations of λ (λ = 0.1, 0.5, 1, 5, and 10) in each language case. The black texts are the average similarity between the velocity vectors in two velocity fields and the p-value for the statistical significance of this average similarity derived from the one-sided permutation test (Permutation Times = 500). The average similarity ranges from 0 to 1, where the value closer to 1 denotes that these two velocity fields are more similar. p-value < 0.05 denotes the statistical significance of the average similarity (significantly not equal to 0). (a1) Each panel demonstrates the distribution within high-dimensional space for the Indo-European language case. (a2) Each panel demonstrates the distribution within high-dimensional space for the Sino-Tibetan language case. (a3) Each panel demonstrates the distribution within high-dimensional space for the Bantu language case. (a4) Each panel demonstrates the distribution within high-dimensional space for the Arawak language case. (b1) Each panel demonstrates the distribution within two-dimensional space for the Indo-European language case. (b2) Each panel demonstrates the distribution within two-dimensional space for the Sino-Tibetan language case. (b3) Each panel demonstrates the distribution within two-dimensional space for the Bantu language case. (b4) Each panel demonstrates the distribution within two-dimensional space for the Arawak language case. We set the default parametric values as k = 10 and m = 1 when varying across the settings of λ. The Source Data and Codes for generating this figure are available.

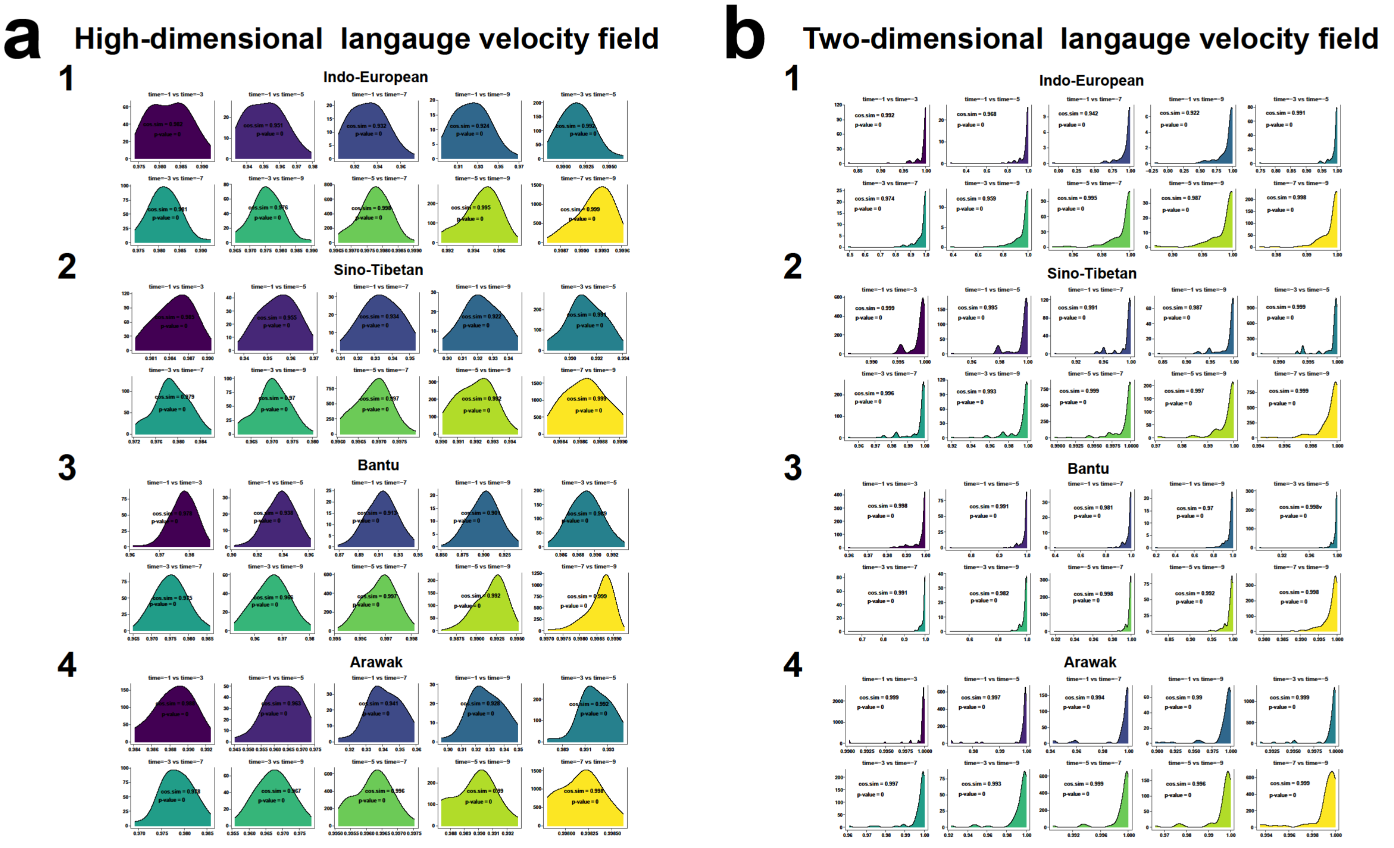

Supplementary Fig. 10: The empirical validations for the robustness of the LVF against the setting of the reconstruction time. Each panel displays a probability density plot that demonstrates the distribution of the cosine similarity between two corresponding velocity vectors in two velocity fields within high-dimensional or two-dimensional spaces estimated under one of the pairwise combinations of reconstruction time m (m = 1, 3, 5, 7, and 9) before the current time in each language case. The black texts are the average similarity between the velocity vectors in two velocity fields and the p-value for the statistical significance of this average similarity derived from the one-sided permutation test (Permutation Times = 500). The average similarity ranges from 0 to 1, where the value closer to 1 denotes that these two velocity fields are more similar. p-value < 0.05 denotes the statistical significance of the average similarity (significantly not equal to 0). (a1) Each panel demonstrates the distribution within high-dimensional space for the Indo-European language case. (a2) Each panel demonstrates the distribution within high-dimensional space for the Sino-Tibetan language case. (a3) Each panel demonstrates the distribution within high-dimensional space for the Bantu language case. (a4) Each panel demonstrates the distribution within high-dimensional space for the Arawak language case. (b1) Each panel demonstrates the distribution within two-dimensional space for the Indo-European language case. (b2) Each panel demonstrates the distribution within two-dimensional space for the Sino-Tibetan language case. (b3) Each panel demonstrates the distribution within two-dimensional space for the Bantu language case. (b4) Each panel demonstrates the distribution within two-dimensional space for the Arawak language case. We set the default parametric values as k = 10 and λ = 1 when varying across the settings of m. The Source Data and Codes for generating this figure are available.

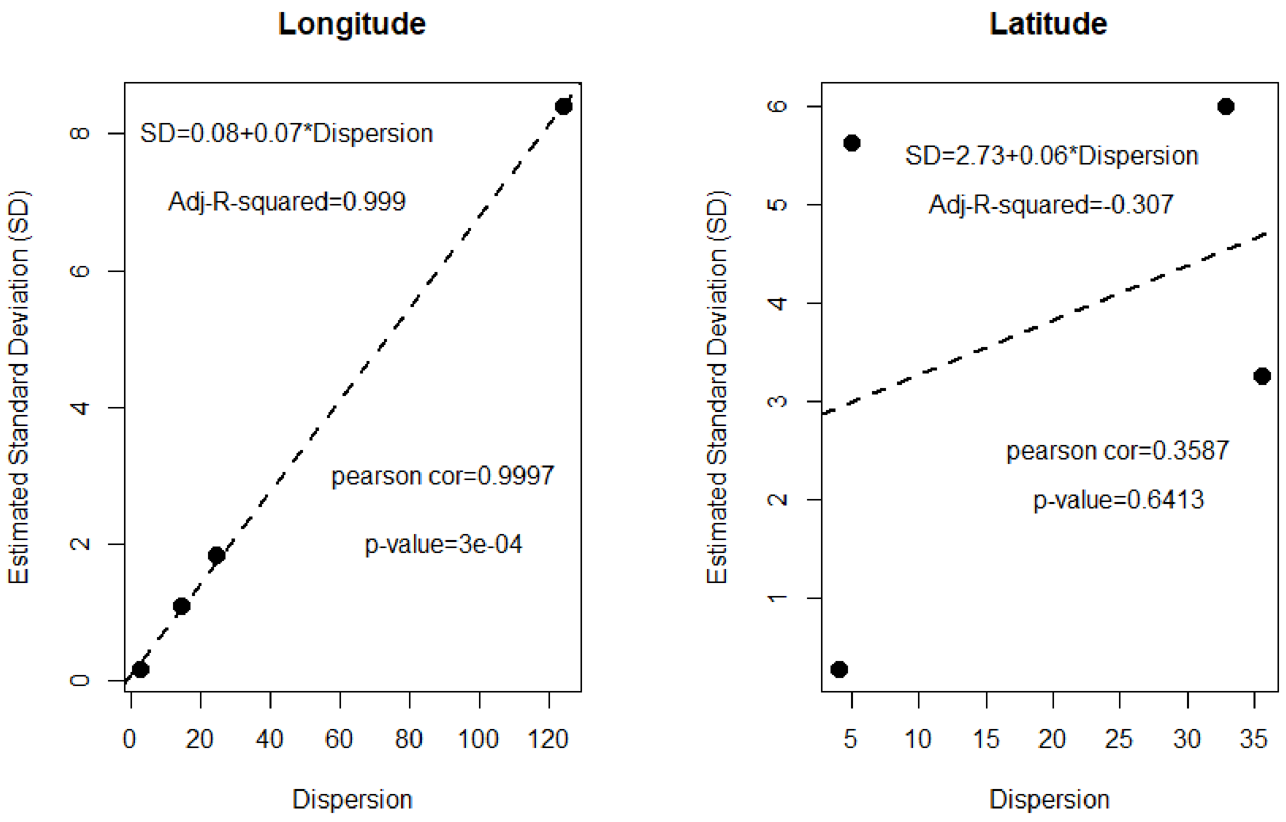

Supplementary Fig. 11: Association between the spatial dispersion and the estimated Standard Deviation (SD) of the geographic coordinate of the language dispersal centre. The black point denotes each language family or group. The dotted line is the regression curve. The texts above the regression curve are the functions of the linear regression model and their adjusted R-squared values. The texts below the regression curve are the person correlation values and *p*-values. The Source Data and Codes for generating this figure are available.

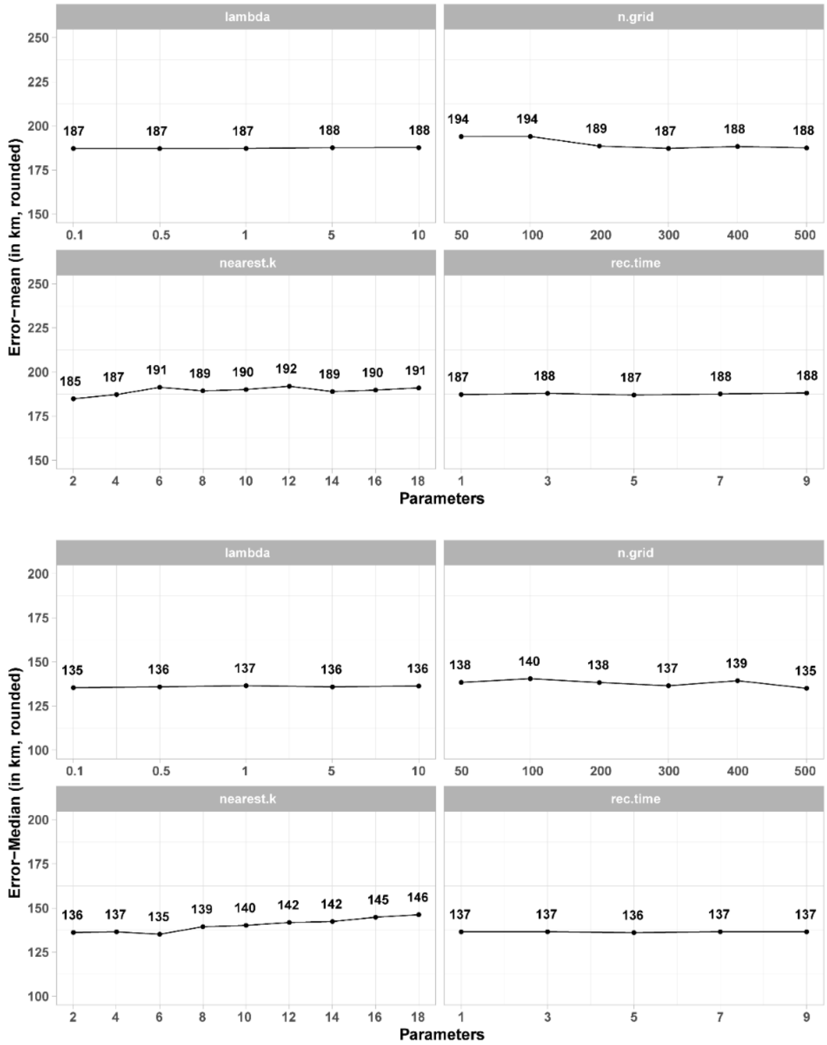

Supplementary Fig. 12: The curves of the estimated errors of LVF under different parametric settings. The line chart demonstrates the variations of the mean and median of 1,000 estimated errors of the inferred coordinates of the language dispersal centre with different parametric settings. The mean and median estimated errors are calculated based on 1,000 simulated datasets and are measured by the great-circle distance (in km, rounded). The parameters are the number of the grid points *n.grid* (*n.grid* = 50, 100, 200, 300, 400, and 500); the number of the nearest neighbours k (k = 2, 4, 6, …, and 18); mutation rate of Poisson process λ (λ = 0.1, 0.5, 1, 5, and 10); reconstruction time m (m = 1, 3, 5, 7, and 9). We set the default parametric values as *n.grid* = 300, k = 4, λ = 1, and m = 1 when varying across the settings of these parameters respectively. The Source Data and Codes for generating this figure are available.
