# Supplementary material for: Inferring language dispersal patterns with velocity field estimation
Source: Nat Commun. 2024 Jan 2;15:190. doi: 10.1038/s41467-023-44430-5 (PMC10761963; doi:10.1038/s41467-023-44430-5)
Supplement: Supplementary file 3 — Reporting Summary [file 41467_2023_44430_MOESM3_ESM.pdf]

Corresponding author(s): Menghan Zhang

Last updated by author(s): Nov 23, 2023

Reporting Summary

Nature Portfolio wishes to improve the reproducibility of the work that we publish. This form provides structure for consistency and transparency in reporting. For further information on Nature Portfolio policies, see our Editorial Policies and the Editorial Policy Checklist.

Statistics

For all statistical analyses, confirm that the following items are present in the figure legend, table legend, main text, or Methods section.

n/a	Confirmed	
☐	☒	The exact sample size (n) for each experimental group/condition, given as a discrete number and unit of measurement
☒	☐	A statement on whether measurements were taken from distinct samples or whether the same sample was measured repeatedly
☐	☒	The statistical test(s) used AND whether they are one- or two-sided<br>*Only common tests should be described solely by name; describe more complex techniques in the Methods section.*
☒	☐	A description of all covariates tested
☐	☒	A description of any assumptions or corrections, such as tests of normality and adjustment for multiple comparisons
☐	☒	A full description of the statistical parameters including central tendency (e.g. means) or other basic estimates (e.g. regression coefficient) AND variation (e.g. standard deviation) or associated estimates of uncertainty (e.g. confidence intervals)
☐	☒	For null hypothesis testing, the test statistic (e.g. F, t, r) with confidence intervals, effect sizes, degrees of freedom and P value noted<br>*Give P values as exact values whenever suitable.*
☒	☐	For Bayesian analysis, information on the choice of priors and Markov chain Monte Carlo settings
☐	☒	For hierarchical and complex designs, identification of the appropriate level for tests and full reporting of outcomes
☒	☐	Estimates of effect sizes (e.g. Cohen's d, Pearson's r), indicating how they were calculated

Our web collection on statistics for biologists contains articles on many of the points above.

Software and code

Policy information about availability of computer code

Data collection	No specific software was used for data collection.
Data analysis	1. The R (4.3.1) software was used for all the analyses which could be downloaded from https://www.r-project.org/.<br>2. We developed a R package namely "LVF" for the implementation of our approach. The package "LVF" and its tutorial are available in GitHub (https://github.com/Stan-Sizhe-Yang/Inferring-language-dispersal-patterns-with-velocity-field-estimation) and Zendo (https://doi.org/10.5281/zenodo.10223872).<br>3. The R codes and all linguistic datasets (including four empirical linguistic datasets and 1,000 simulated datasets) used in this study are available in GitHub (https://github.com/Stan-Sizhe-Yang/Inferring-language-dispersal-patterns-with-velocity-field-estimation) and Zendo (https://doi.org/10.5281/zenodo.10223872).

For manuscripts utilizing custom algorithms or software that are central to the research but not yet described in published literature, software must be made available to editors and reviewers. We strongly encourage code deposition in a community repository (e.g. GitHub). See the Nature Portfolio guidelines for submitting code & software for further information.

Data

Policy information about availability of data

All manuscripts must include a data availability statement. This statement should provide the following information, where applicable:

- Accession codes, unique identifiers, or web links for publicly available datasets
- A description of any restrictions on data availability
- For clinical datasets or third party data, please ensure that the statement adheres to our policy

The data supporting the findings of this study are available in GitHub (https://github.com/Stan-Sizhe-Yang/Inferring-language-dispersal-patterns-with-velocity-field-estimation) and Zendo (https://doi.org/10.5281/zenodo.10223872).

Research involving human participants, their data, or biological material

Policy information about studies with human participants or human data. See also policy information about sex, gender (identity/presentation), and sexual orientation and race, ethnicity and racism.

Reporting on sex and gender	No relevant to this study.
Reporting on race, ethnicity, or other socially relevant groupings	No relevant to this study.
Population characteristics	No relevant to this study.
Recruitment	No relevant to this study.
Ethics oversight	No relevant to this study.

Note that full information on the approval of the study protocol must also be provided in the manuscript.

Field-specific reporting

Please select the one below that is the best fit for your research. If you are not sure, read the appropriate sections before making your selection.

☒ Life sciences ☐ Behavioural & social sciences ☐ Ecological, evolutionary & environmental sciences

For a reference copy of the document with all sections, see nature.com/documents/nr-reporting-summary-flat.pdf

Life sciences study design

All studies must disclose on these points even when the disclosure is negative.

Sample size	In this study, we used 692 language samples of four language families and groups. They are 103 Indo-European languages, 109 Sino-Tibetan languages, 420 Bantu languages, and 60 Arawak languages, respectively. We also collated their geographic coordinates of longitude and latitude. According to the available linguistic data of these 692 languages, there are total 11,496 binary-coded lexical cognates involved in this study. The detailed description can be found in the "Linguistic data" section of Methods, and linguistic data are available in GitHub (https://github.com/Stan-Sizhe-Yang/Inferring-language-dispersal-patterns-with-velocity-field-estimation) and Zendo (https://doi.org/10.5281/zenodo.10223872).<br><br>We also performed several simulations to evaluate the effectiveness and robustness of our approach using 1,000 simulated datasets derived from Wichmann and Rama (2021), of which the dispersal centers were known. Each simulated dataset consisted of 20 language samples with the same 306 binary-coded traits, in which each language had a different coordinate. The details can be found in Methods, and Supplementary Notes section 2. The simulated linguistic data are available in GitHub (https://github.com/Stan-Sizhe-Yang/Inferring-language-dispersal-patterns-with-velocity-field-estimation) and Zendo (https://doi.org/10.5281/zenodo.10223872).
Data exclusions	Due to our approach does not allow missing values as input, we filtered out linguistic traits with missing values > 75% and performed the mod-value imputation strategy to impute the missing values of the rest of the linguistic traits.
Replication	We conducted several simulated validations based on additional 1,000 simulation datasets and numerous empirical validations based on four realistic datasets to validate the robustness and efficiency of the language velocity field estimation approach. Also, we replicated our validations with different parametric settings and missing-value imputation strategies. The replication results were illustrated in Supplementary Figures 2, 3,7,8,9, and 10, and Supplementary Table 3. The detailed processes of validations can found in Supplementary Methods and Supplementary Notes.
Randomization	This is not relevant to our study because our study is purely computational that does not involve any experiments.
Blinding	This is not relevant to our study because our study is purely computational that does not involve any experiments.

Reporting for specific materials, systems and methods

We require information from authors about some types of materials, experimental systems and methods used in many studies. Here, indicate whether each material, system or method listed is relevant to your study. If you are not sure if a list item applies to your research, read the appropriate section before selecting a response.

Materials & experimental systems

n/a	Involved in the study
☒	☐ Antibodies
☒	☐ Eukaryotic cell lines
☒	☐ Palaeontology and archaeology
☒	☐ Animals and other organisms
☒	☐ Clinical data
☒	☐ Dual use research of concern
☒	☐ Plants

Methods

n/a	Involved in the study
☒	☐ ChIP-seq
☒	☐ Flow cytometry
☒	☐ MRI-based neuroimaging
